# Supplementary material for: Temporally programmed polymer – solvent interactions using a chemical reaction network
Source: Nat Commun. 2022 Oct 21;13:6242. doi: 10.1038/s41467-022-33810-y (PMC9587023; doi:10.1038/s41467-022-33810-y)
Supplement: Supplementary file 1 — Supplementary Information [file 41467_2022_33810_MOESM1_ESM.pdf]

Supplementary Information

Temporally programmed polymer - solvent interactions using a chemical reaction network

Benjamin Klemm[a], Reece W. Lewis[a], Irene Piergentili[a], and Rienk Eelkema[a,*]

[a] Department of Chemical Engineering, Delft University of Technology, Van der Maasweg 9, 2629 HZ Delft, The Netherlands.

* Correspondence to: R.Eelkema@tudelft.nl

Table of Contents

Supplementary Notes **3**

Instrumentation and materials **3**

Supplementary Figures and Tables **4**

1.0 Fluorescence measurements **4**

2.0 Kinetic experiments **4**

2.1 Summary of reaction rate constants 5

2.2 Kinetic experiments – Blank reactions 6

2.3 Kinetic experiments – signal induced cycle 8

2.4 Kinetic experiments – autonomous cycle 10

3.0 Micelle assembly/disassembly study **13**

3.1 Micelle study – signal induced cycle 13

3.2 Micelle study – autonomous cycle 14

3.3 Micelle study - DLS intensity and number% average 15

4.0 Time-lapse observation for hydrogel experiments **17**

5.0 Synthetic procedures **19**

Synthesis of p($4VP_{58}$-b-DMA_{261}) – polymer for micelles (P1) 19

Synthesis of p($4VP_{28}$-stat-DMA_{55}) – P2 precursor used for hydrogel preparation 21

Synthesis of diethyl(α-acetoxymethyl) vinylphosphonate (DVP) 22

Synthesis of 1-(2-(diethoxyphosphoryl)allyl)-1,4 diazabicyclo[2.2.2] 23

octanium chloride (DVP-t-Am-1) 23

Synthesis of 1-(2-(diethoxyphosphoryl)allyl)pyridinium chloride 23

(DVP-t-Am-2) 23

Synthesis of 3-(2-hydroxyethylsulfanyl)prop-1-en-2-ylphosphonate 24

(DVP-S) 24

Synthesis of 2-((2-(diethoxyphosphoryl)allyl)amino)-3-hydroxybutanoic acid (DVP-N) 24

6.0 NMR Spectra **25**

7.0 LC-MS data **34**

Supplementary References **40**

Supplementary Notes

Instrumentation and materials

All reagents and solvents were used without further purification unless otherwise stated. Tetraethylmethylene diphosphonate (TMP, 97%), para-formaldehyde (p-FA, 95%) 2-mercaptoethanol (SH-**3**, >99%), N,N-dimethylacrylamide (DMA, 99%) , 4-vinylpyridine (4VP, 95%), 4-((((2-carboxyethyl)thio)carbonothioyl)thio)-4-cyanopentanoic acid (CETCPA, 95%, ABCR), 3-(trimethylsilyl)-1-propanesulfonic acid sodium salt (DSS, 97%), *N,N'*-methylenebis(acrylamide) (BisAM, 97%), acetic anhydride (AA, >99%), dimethylaminopyridine (DMAP, >99%), triethylamine (Et_3N, 98%), L-Threonine (NH_2-**4,** >98%), 1,4-diazabicyclo[2.2.2]octane - DABCO (t-Am-**1,** >99%), pyridine (t-Am-**2,** >99%), tetramethylethylenediamine (TEMED, 99%) and ammonium persulfate (APS, 98%) were purchased from Sigma Aldrich or TCI Europe. For the preparation of aqueous-buffers, solid salts were used: sodium phosphate monobasic and sodium phosphate dibasic which were purchased from Sigma Aldrich. Unless stated otherwise, all stock solutions were prepared in D_2O/phosphate buffer mixtures 1:9 (0.1 M, pH = 7.4) for the signal-induced cycle and D_2O/phosphate buffer mixtures 1:9 (0.5 M, pH = 7.4) for the autonomous cycle experiments to avoid significant pH changes. All buffers were pH adjusted using sodium hydroxide (1 M) and hydrochloric acid (1 M). DMA and 4VP were passed through basic alumina prior to use to remove inhibitor. ESI-MS was performed using LTQ XL spectrometer equipped with Shimadzu HPLC setup operating at 0.2 mL/min flow rate with water/MeCN mobile phase containing 0.1 vol% formic acid and Discovery C18 column. Dynamic Light Scattering (DLS) measurements were performed on a Malvern Instruments Zetasizer Nano ZS, employing a 633 nm laser at a back-scattering angle of 173°. Measurements were performed in BRAND 1.5 mL PMMA semi-micro disposable cuvette (10 mm path length). TEM measurements were performed on a Jeol JEM1400 Transmission Electron Microscope with an operating voltage of 120kV. Fluorescence spectra were recorded with a fluorescence spectrometer Spex Fluorolog-3 equipped with a standard 90° setup, operated at a constant Voltage of 25 V. Photographs of the hydrogels were taken on a Canon EOS 600D single reflex camera with a Canon Macro Lens EF 100 mm 1:2.8 USM. The LED reactor was constructed from a 5-meter strip of 300 RGB 5050 SMD LEDs procured from ryslux (ebay) with λ_{max} of the blue lights measured to be 441 nm (Ocean Optics USB 4000 fibre coupled spectrometer). These LEDs were connected around a glass beaker of diameter 10 cm.

Supplementary Figures and Tables

1.0 Fluorescence measurements

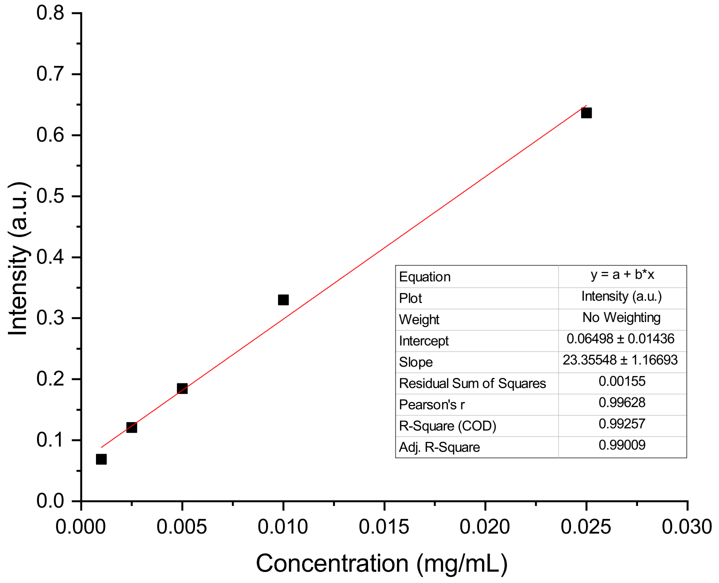

Supplementary Figure 1: Calibration curve - Nile Red concentration (mg/mL) vs. fluorescence intensity (-).

2.0 Kinetic experiments

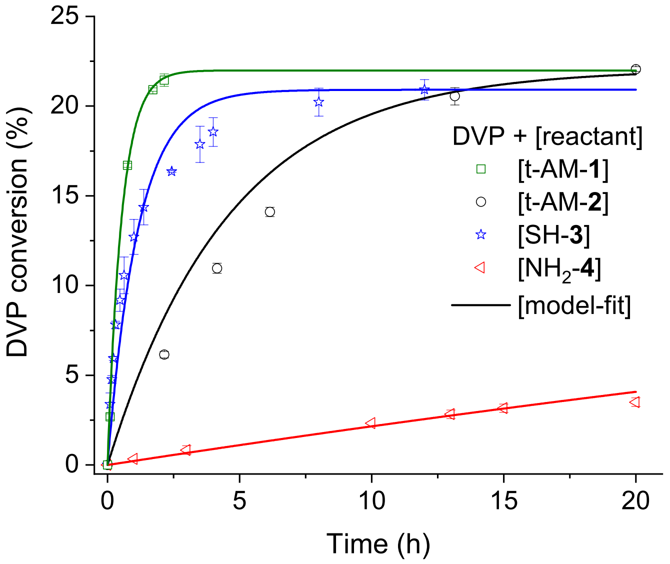

Supplementary Figure 2: Conversion plot over time for the reaction of DVP (1.0 eq.) with t-AM-1 (0.2 eq.), t-AM-2 (0.2 eq.), SH-3 (0.2 eq.) or NH_2-4 (0.2 eq.). All measurements were done in duplicate. The error bars represent the standard deviation of duplicate measurements. Solid lines represent the k-value-model fit to the experimental data.

2.1 Summary of reaction rate constants

Supplementary Table 1: Summary of the reaction rates for DVP with t-AM-1, t-AM-2, SH-3 or NH_2-4.

A

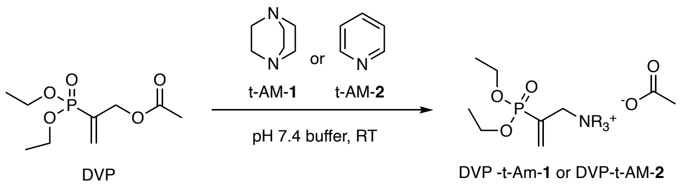

B

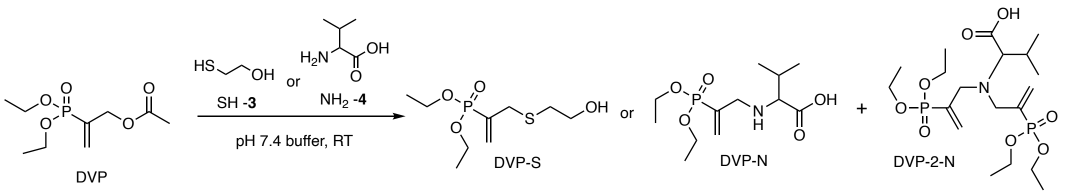

Scheme	Reaction	k (M^{-1}*h^{-1})	R^2
A	DVP + t-AM-**1**	43.5 ± 3.21	0.996
	DVP + t-AM-**2**	5.14 ± 0.62	0.992
B	DVP + SH-**3**	20.3 ± 4.74	0.982
	DVP + NH_2-**4**	0.27 ± 0.02	0.995

*** Conditions: (A) 0.04 mM of DVP, 0.008 mM t-AM-1 or t-AM-2 in 0.1 M phosphate buffer (pH 7.4), 25 °C, (B) 0.04 mM of DVP, 0.008 mM of nucleophile (SH-3 or NH_2-4) in 0.1 M phosphate buffer (pH 7.4), 25 °C.**

2.2 Kinetic experiments – Blank reactions

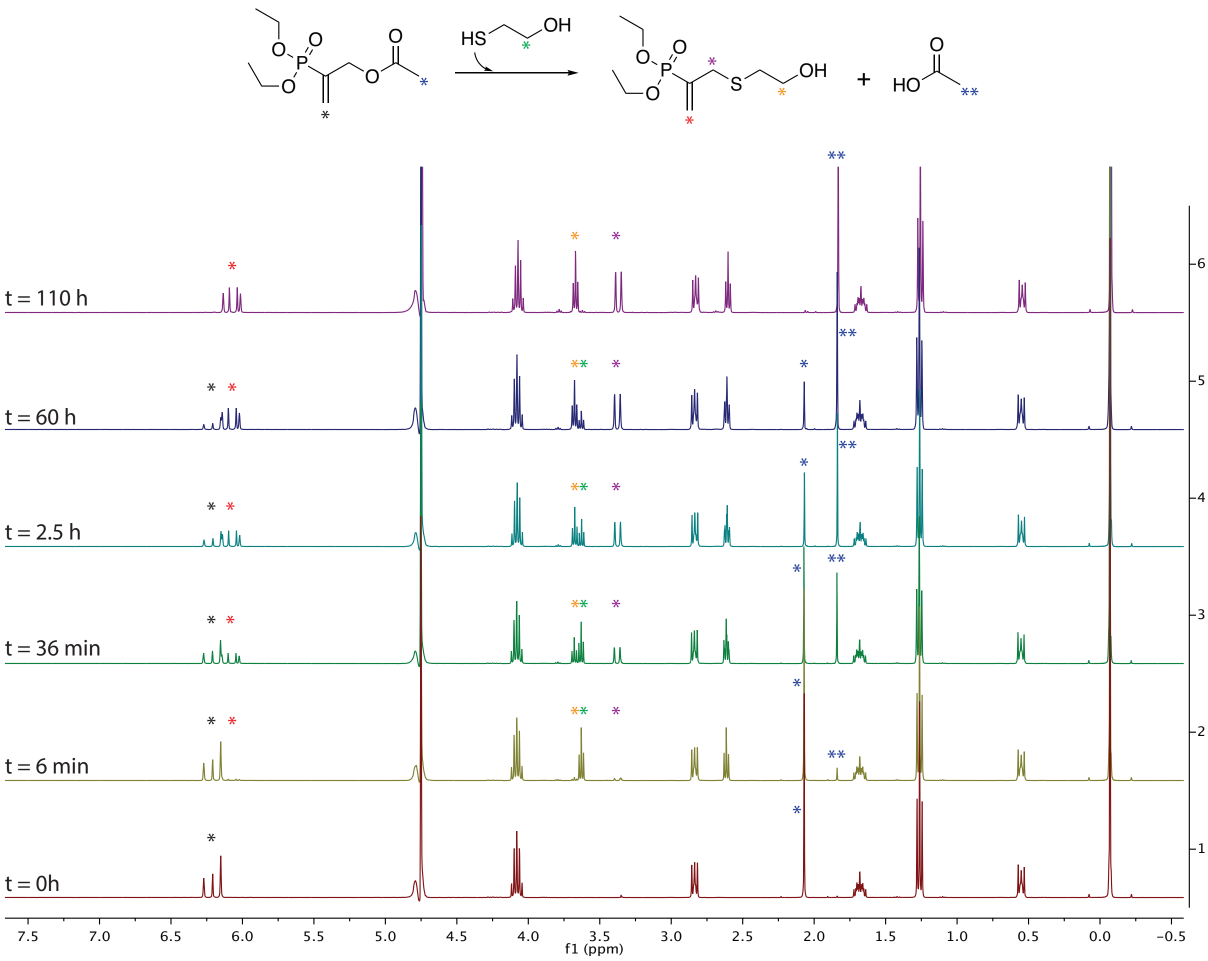

Supplementary Figure 3: Reaction spectra of DVP with SH-3 followed by ^{1}H NMR at different time points. The reaction was carried out in D_2O/phosphate buffer mixture 1:9 (0.1 M, pH = 7.4). The spectra were aligned with D_2O peak. The peak attributed to ~ 0.0 ppm corresponds to DSS internal standard.

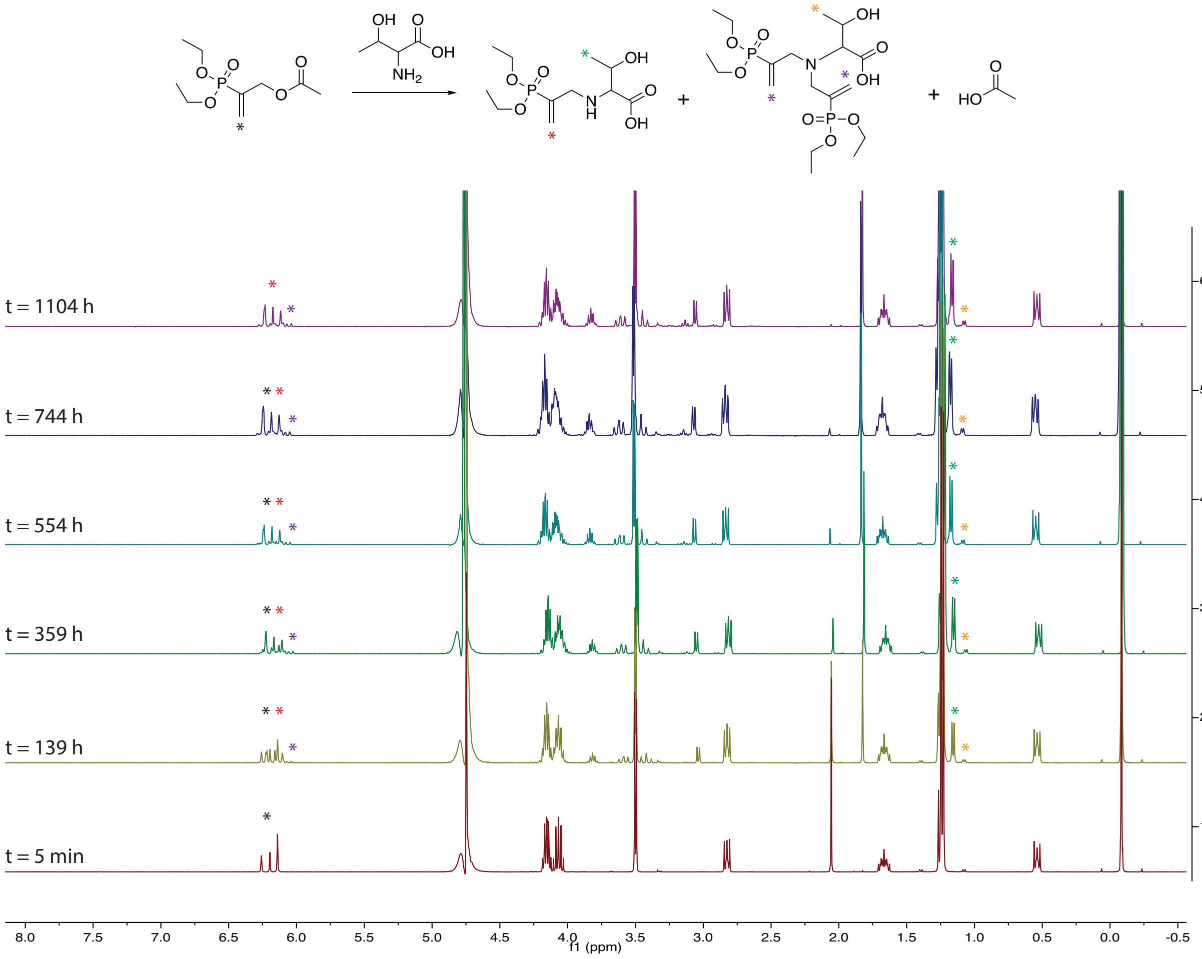

Supplementary Figure 4: Reaction spectra of DVP with NH_2-4 followed by ^1H NMR at different time points. The reaction was carried out in D_2O/phosphate buffer mixture 1:9 (0.5 M, pH = 7.4). The spectra were aligned with D_2O peak. The peak at ~ 0.0 ppm corresponds to DSS internal standard.

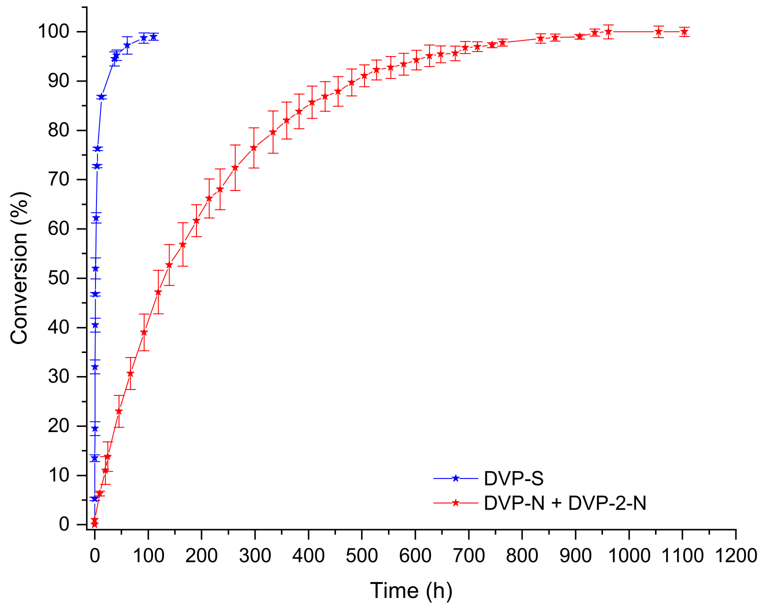

Supplementary Figure 5: Conversion plot over time for the reaction of DVP (1.0 eq.) with SH-3 (1.0 eq.) and DVP (1.0 eq.) with NH_2-4 (4.0 eq.) in duplicate, respectively. The error bars represent the standard deviation of duplicate measurements.

2.3 Kinetic experiments – signal induced cycle

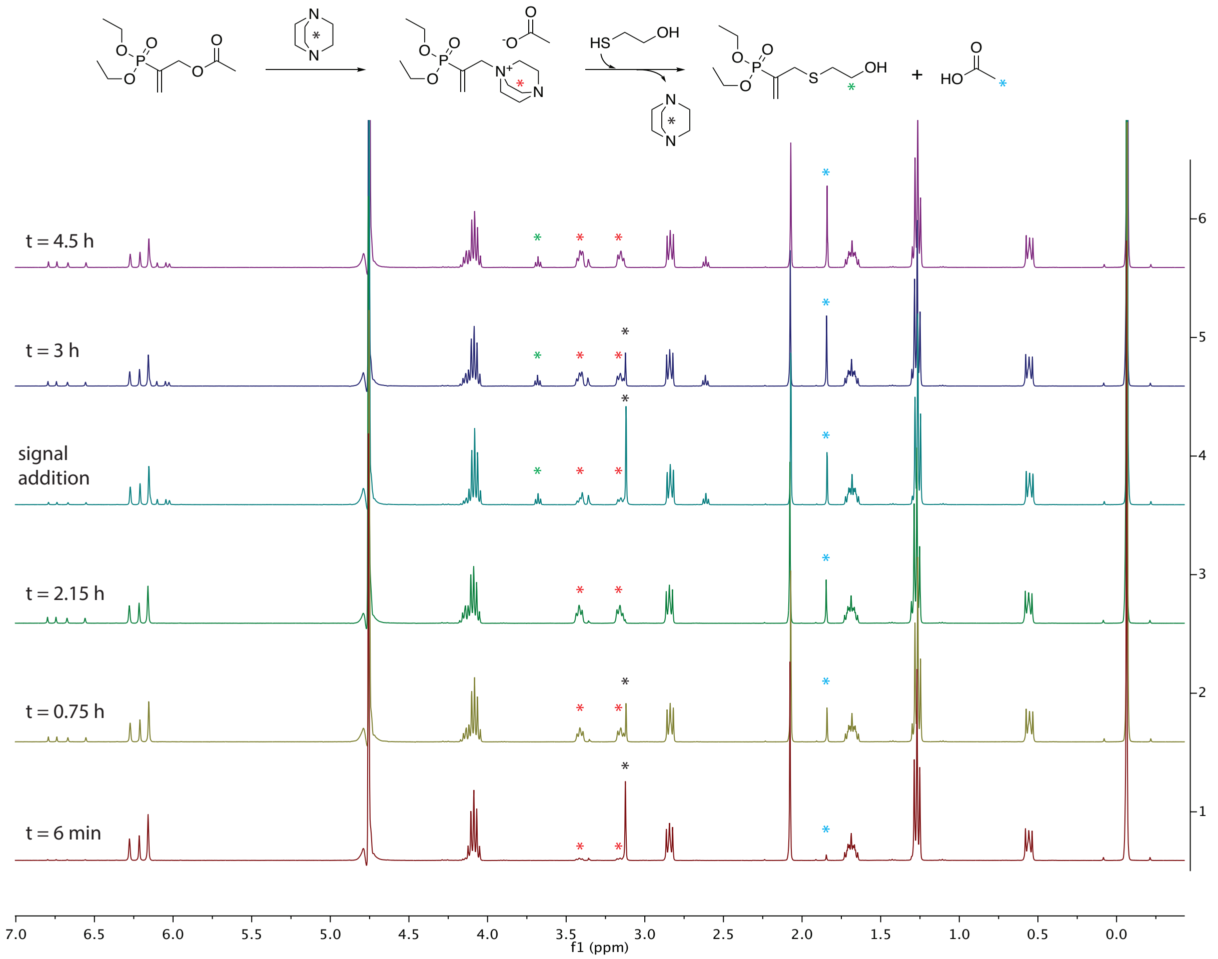

Supplementary Figure 6: Reaction (signal-induced cycle) of DVP, t-Am-1 with signal addition of SH-3 followed by ^{1}H NMR at different time points. At t = 0 h: DVP and t-Am-1 are added and followed by ^{1}H NMR upon complete formation of DVP–t-Am-1 (t = 2.15 hours), then SH-3 in equimolar amounts is added (signal addition) and the reaction is continuously followed by ^{1}H NMR until one full cycle was achieved (t = 4.5 hours). The reaction was carried out in D_2O/phosphate buffer mixture 1:9 (0.1 M, pH = 7.4). The spectra were aligned with D_2O peak. The peak at ~ 0.0 ppm corresponds to DSS internal standard.

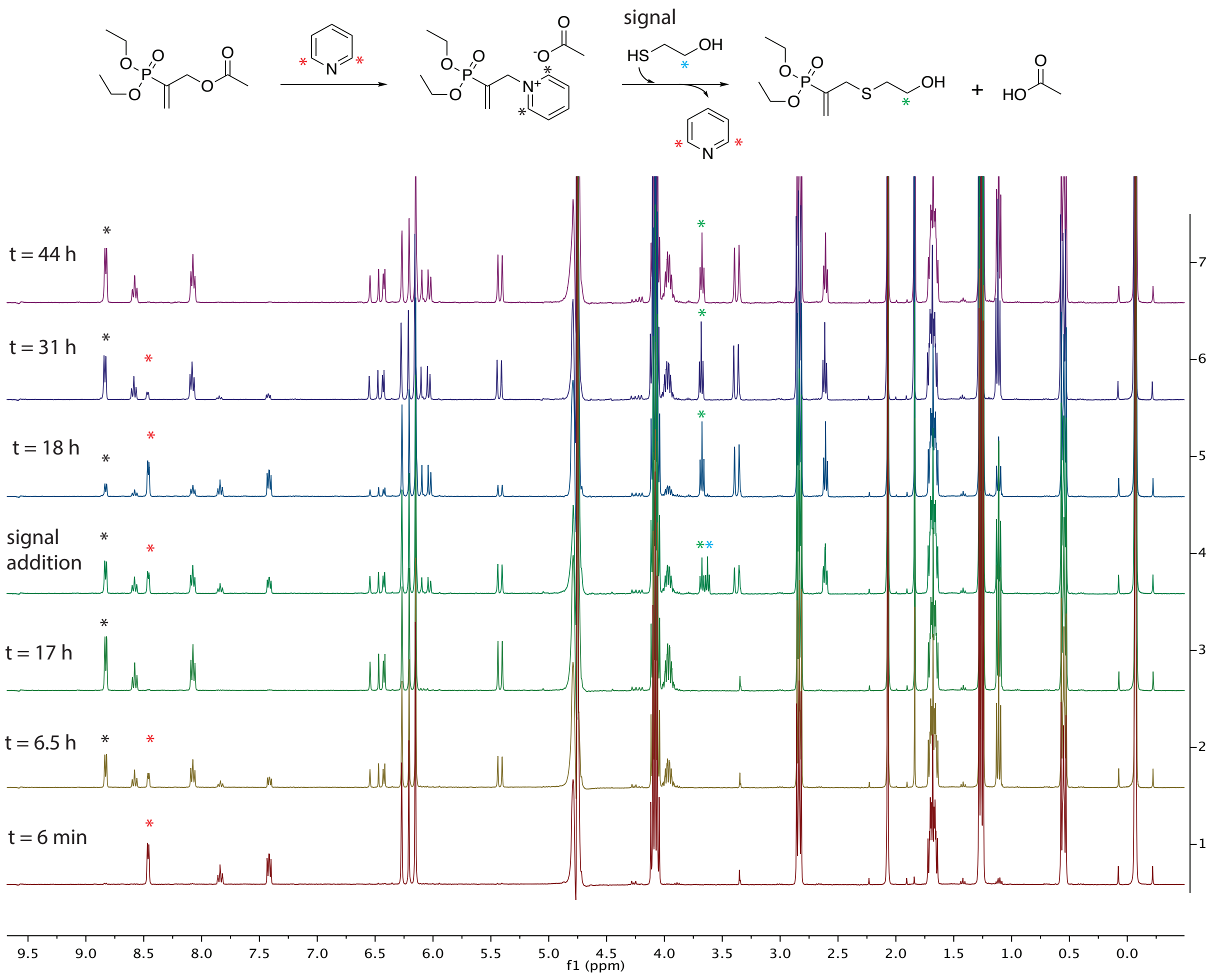

Supplementary Figure 7: Reaction (signal-induced cycle) of DVP, t-Am-2 with signal addition of SH-3 followed by ^{1}H NMR at different time points. At t = 0 h: DVP and t-Am-2 are added and followed by ^{1}H NMR upon complete formation of DVP-t-Am-2 (t = 17 hours), then SH-3 in equimolar amounts is added (signal addition) and the reaction is continuously followed by ^{1}H NMR until one full cycle was achieved (t = 44 hours). The reaction was carried out in D_2O/phosphate buffer mixture 1:9 (0.1 M, pH = 7.4). The spectra were aligned with D_2O peak. The peak at ~ 0.0 ppm corresponds to DSS internal standard.

2.4 Kinetic experiments – autonomous cycle

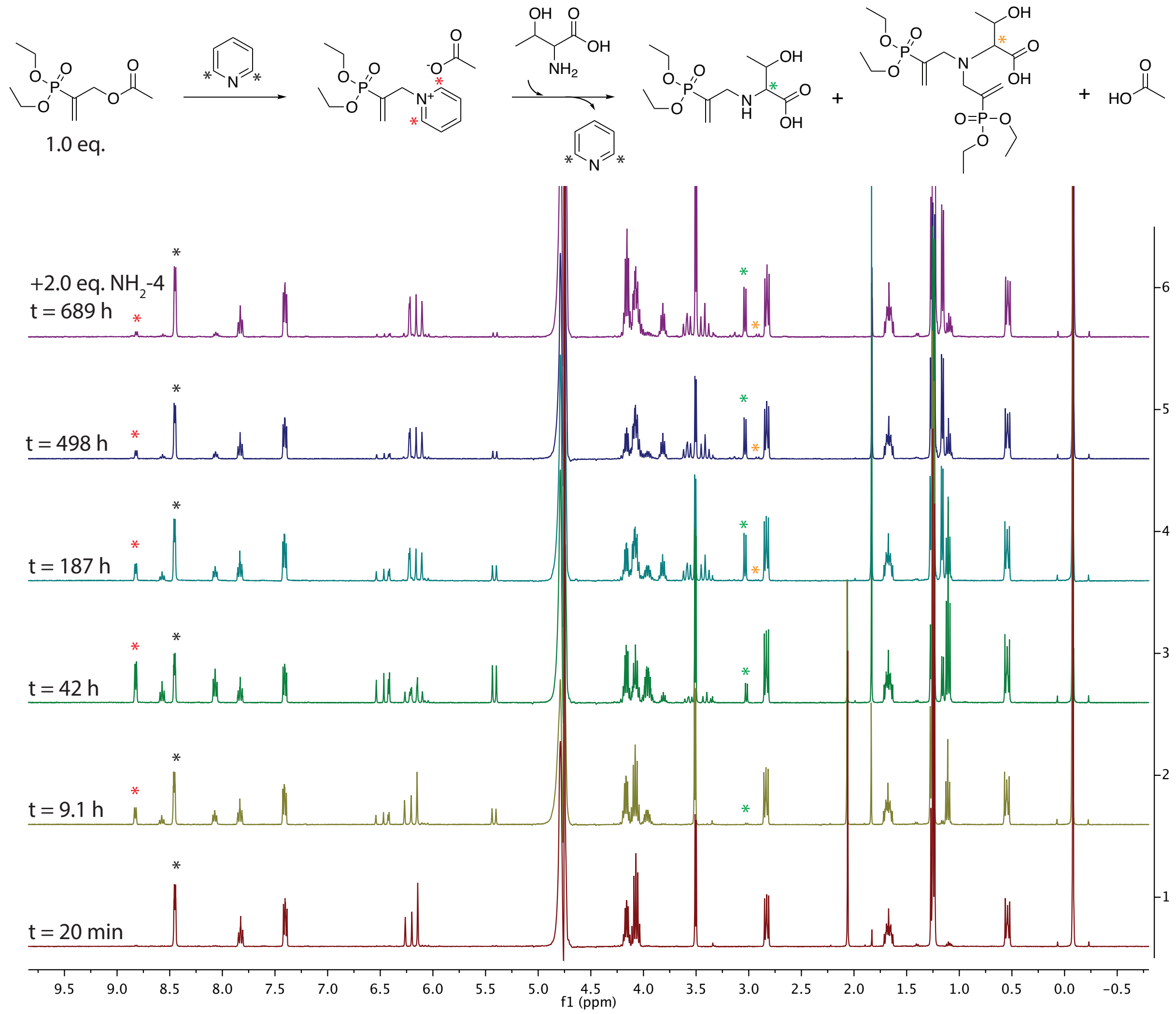

Supplementary Figure 8: Reaction of DVP (1.0 eq.), t-Am-2 (1.0 eq.) with NH_2-4 (2.0 eq.) followed by ^1H NMR at different time points. The reaction was carried out in D_2O/phosphate buffer mixture 1:9 (0.5 M, pH = 7.4). The spectra were aligned with D_2O peak. The peak at ~ 0.0 ppm corresponds to DSS internal standard.

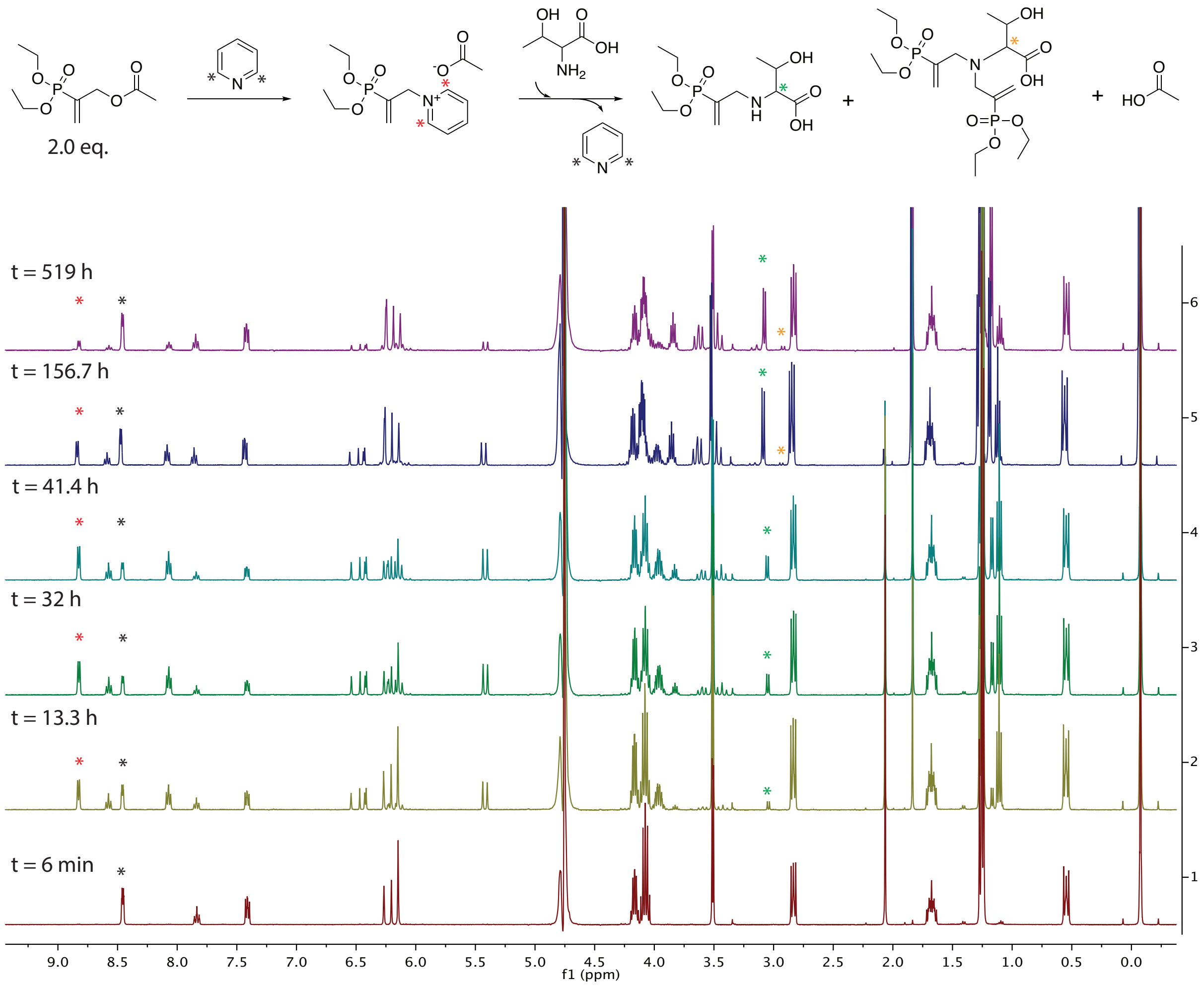

Supplementary Figure 9: Reaction of DVP (2.0 eq.), t-Am-2 (1.0 eq.) with NH_2-4 (4.0 eq.) followed by ^1H NMR at different time points. The reaction was carried out in D_2O/phosphate buffer mixture 1:9 (0.5 M, pH = 7.4). The spectra were aligned with D_2O peak. The peak at ~ 0.0 ppm corresponds to DSS internal standard.

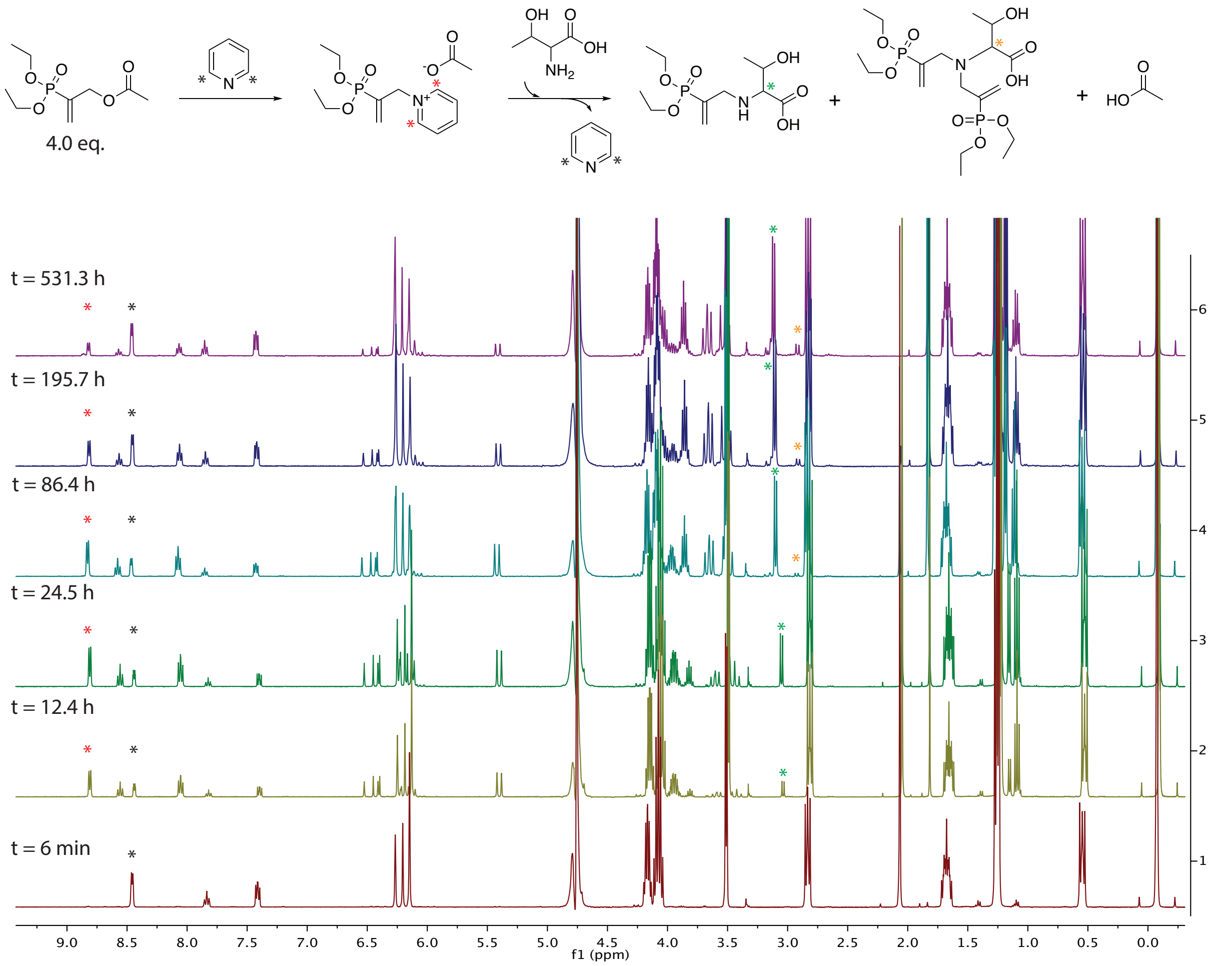

Supplementary Figure 10: Reaction of DVP (4.0 eq.), t-Am-2 (1.0 eq.) with NH_2-4 (8.0 eq.) followed by ^1H NMR at different time points. The reaction was carried out in D_2O/phosphate buffer mixture 1:9 (0.5 M, pH = 7.4). The spectra were aligned with D_2O peak. The peak at ~ 0.0 ppm corresponds to DSS internal standard.

3.0 Micelle assembly/disassembly study

3.1 Micelle study – signal induced cycle

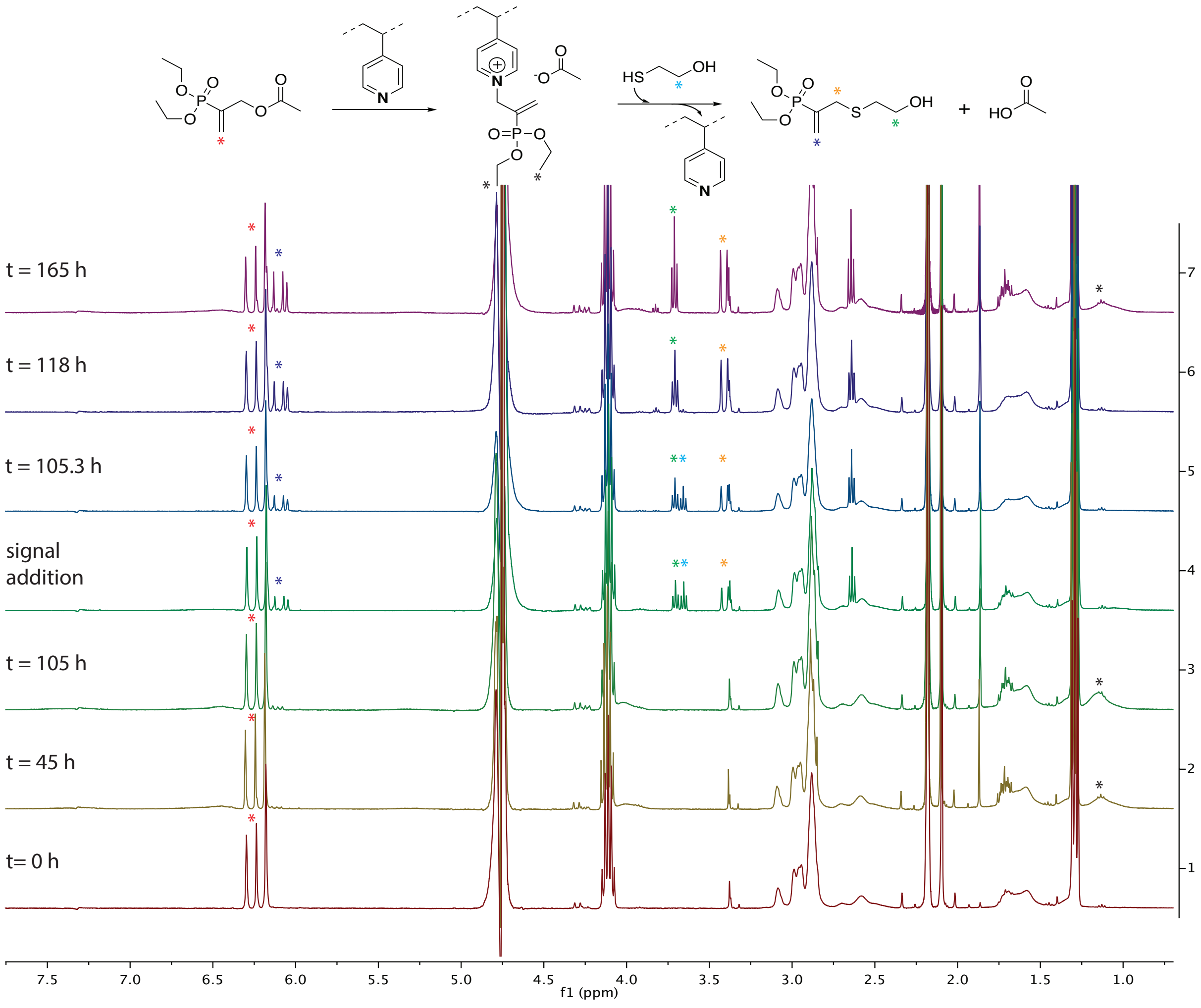

Supplementary Figure 11: Reaction of DVP (3.2 eq.), P1 (1.0 eq. 4VP) with signal addition of SH-3 followed by ^{1}H NMR at different time points. At t = 0: DVP and P1 are added and followed by ^{1}H NMR upon complete formation of DVP - polymer intermediate (t = 105 hours), then SH-3 in equimolar amounts is added (signal addition) and the reaction is continuously followed by ^{1}H NMR until one full cycle is achieved (t = 165 hours). The reaction was carried out in D_2O/phosphate buffer mixture 1:9 (0.1 M, pH = 7.4). The spectra were aligned with the D_2O peak.

3.2 Micelle study – autonomous cycle

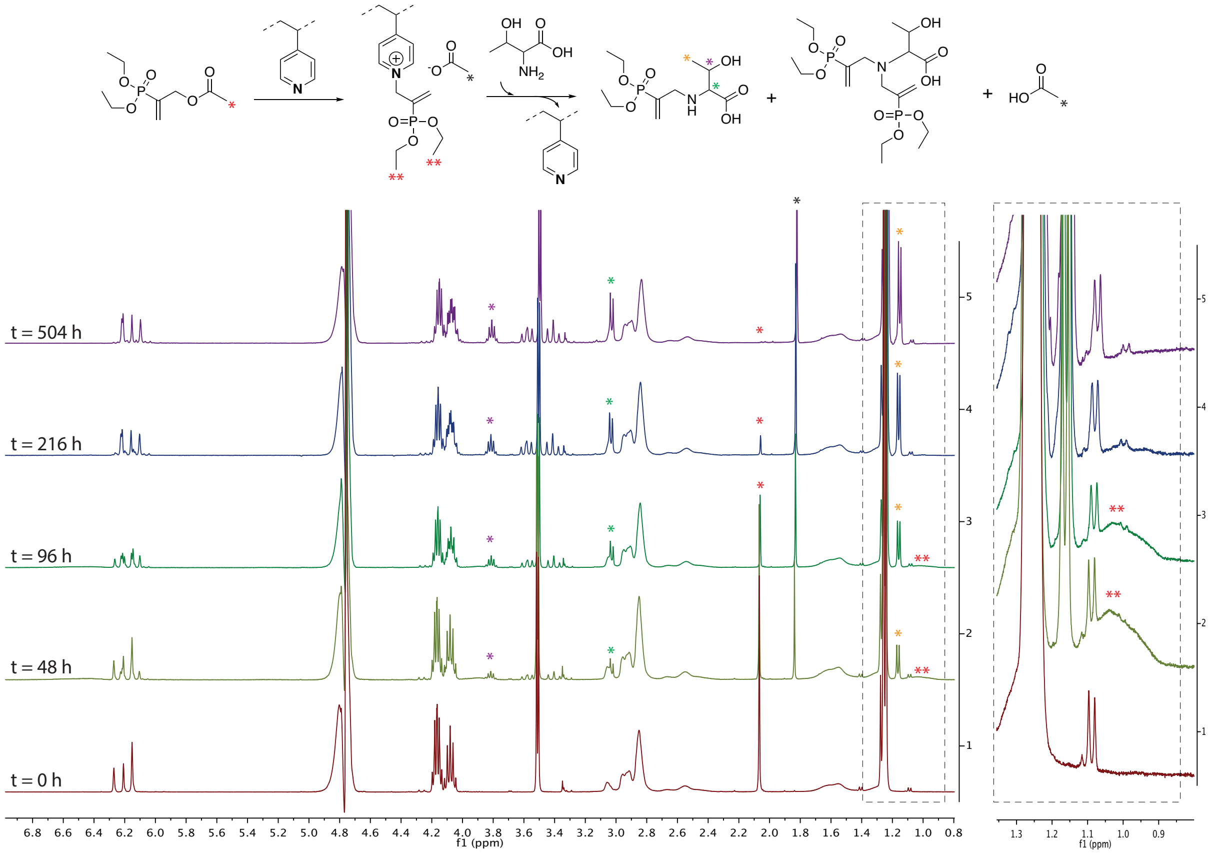

Supplementary Figure 12: Reaction of DVP (2.0 eq.), P1 (1.0 eq. 4VP) with NH_2-4 (8.0 eq.) followed by ^1H NMR at different time points. At t = 0: DVP, NH_2-4 and P1 are added and followed by ^1H NMR. One full cycle is achieved within 504 hours. The reaction was carried out in D_2O/phosphate buffer mixture 1:9 (0.5 M, pH = 7.4). The spectra were aligned with the D_2O peak.

3.3 Micelle study - DLS intensity and number% average

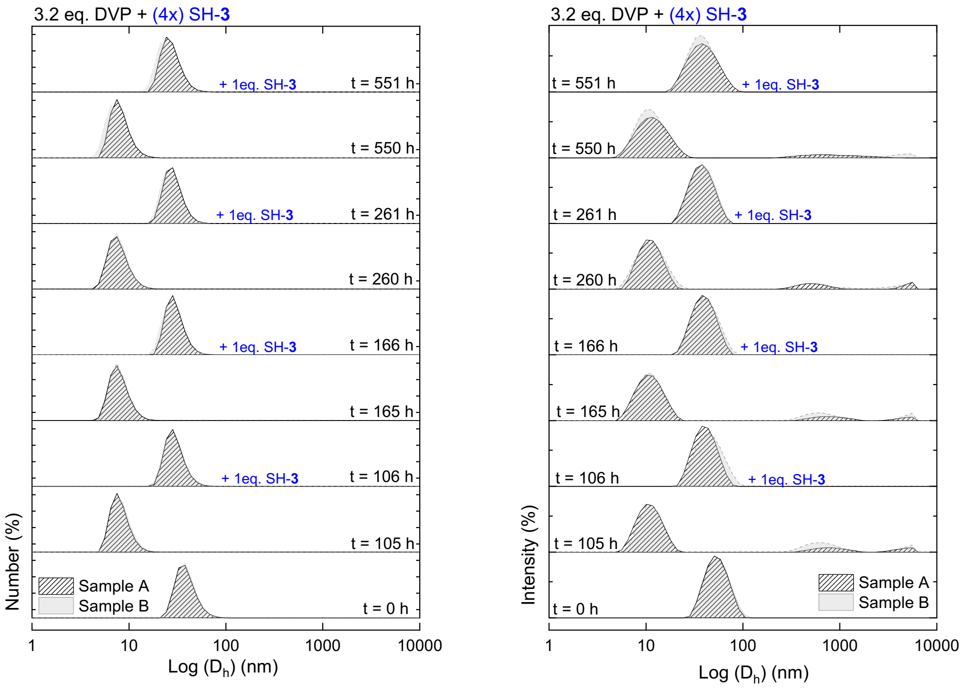

Supplementary Figure 13: DLS data for signal-induced cycle (right) intensity plot over time with micellar size (nm) change upon signal addition (blue: 1.0 eq. SH-3), (left) number% average change over time upon signal addition. Measurements are shown in duplicate (Sample A&B).

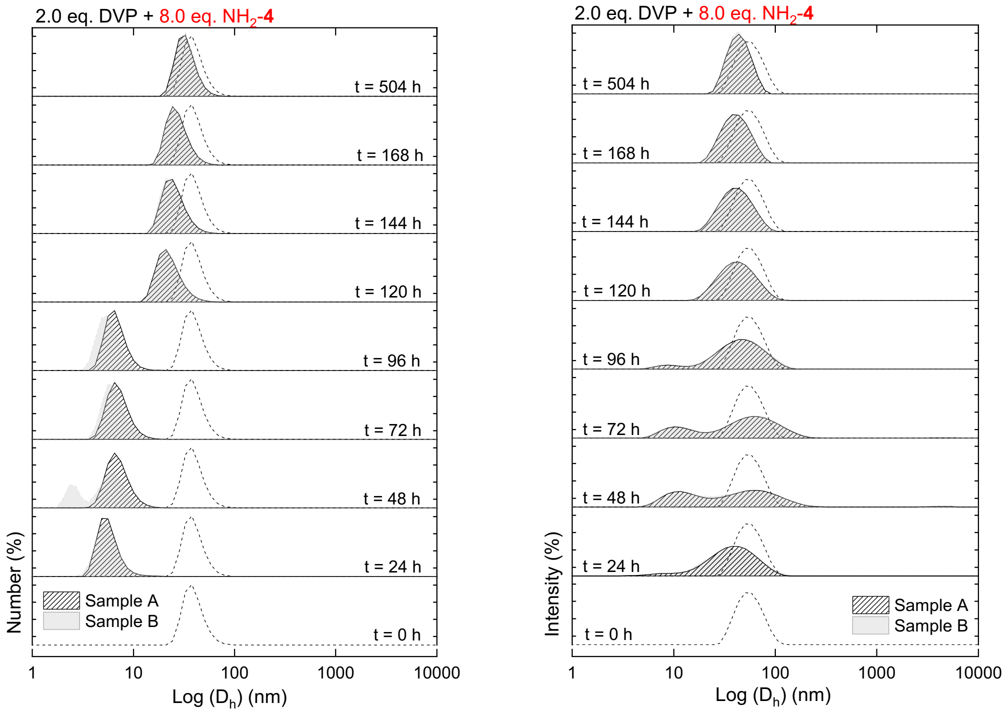

Supplementary Figure 14: DLS data for autonomous cycle (right) intensity plot over time with micellar size (nm) change, (left) number% average change over time. Measurements are shown in duplicate (Sample A&B). The dotted line in the time measurements represents the t = 0 measurement and is shown for comparison.

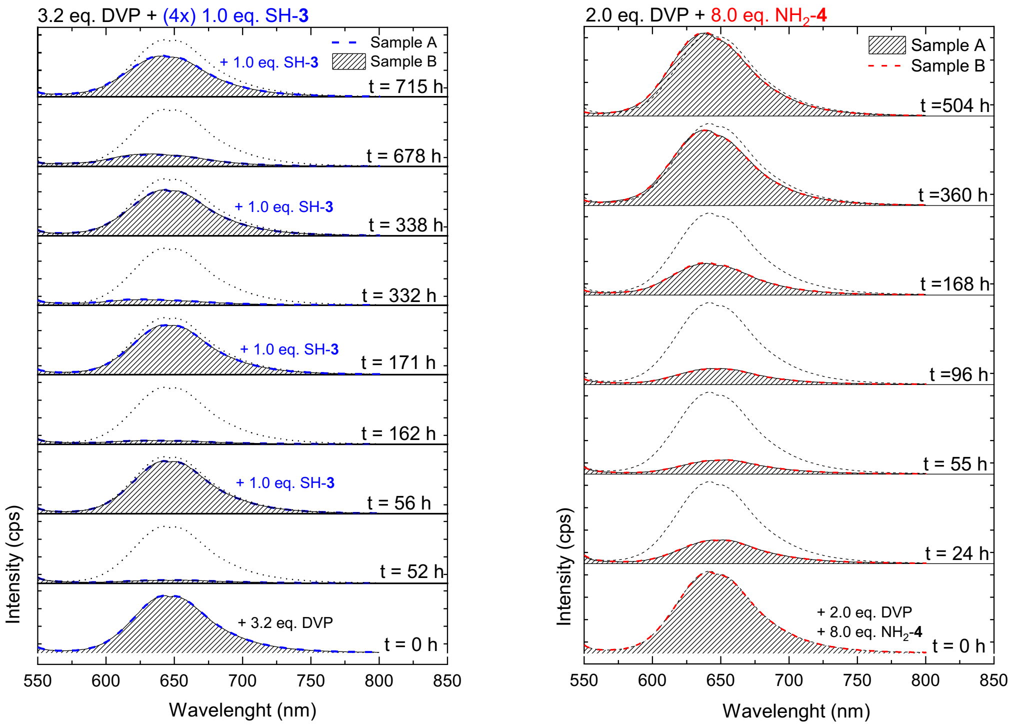

Supplementary Figure 15: Fluorescence spectroscopy intensity plot for autonomous cycle (right) and signal-induced cycle (left) over time. Measurements are shown in duplicate (Sample A&B). The dotted line in the time measurements represents the t = 0 measurement and is shown for comparison.

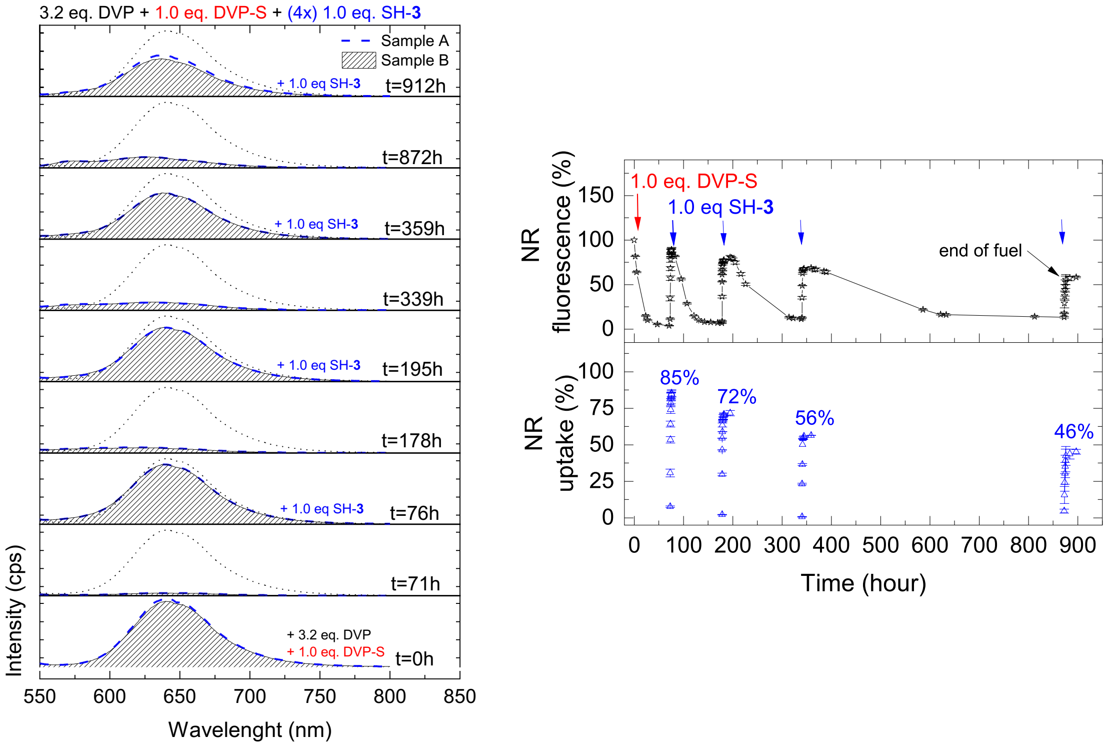

Supplementary Figure 16: Fluorescence spectroscopy intensity plot for signal-induced cycle with 1.0 eq. of waste (DVP-S) added (right) and micellar (dis)assembly with corresponding dye uptake profile followed by NR fluorescence (left) at an excitation wavelength of 540 nm and an emission wavelength of 645 nm. Measurements are shown in duplicate (Sample A&B). The error bars represent the standard deviation of duplicate measurements. The dotted line in the time measurements represents the t = 0 measurement and is shown for comparison.

4.0 Time-lapse observation for hydrogel experiments

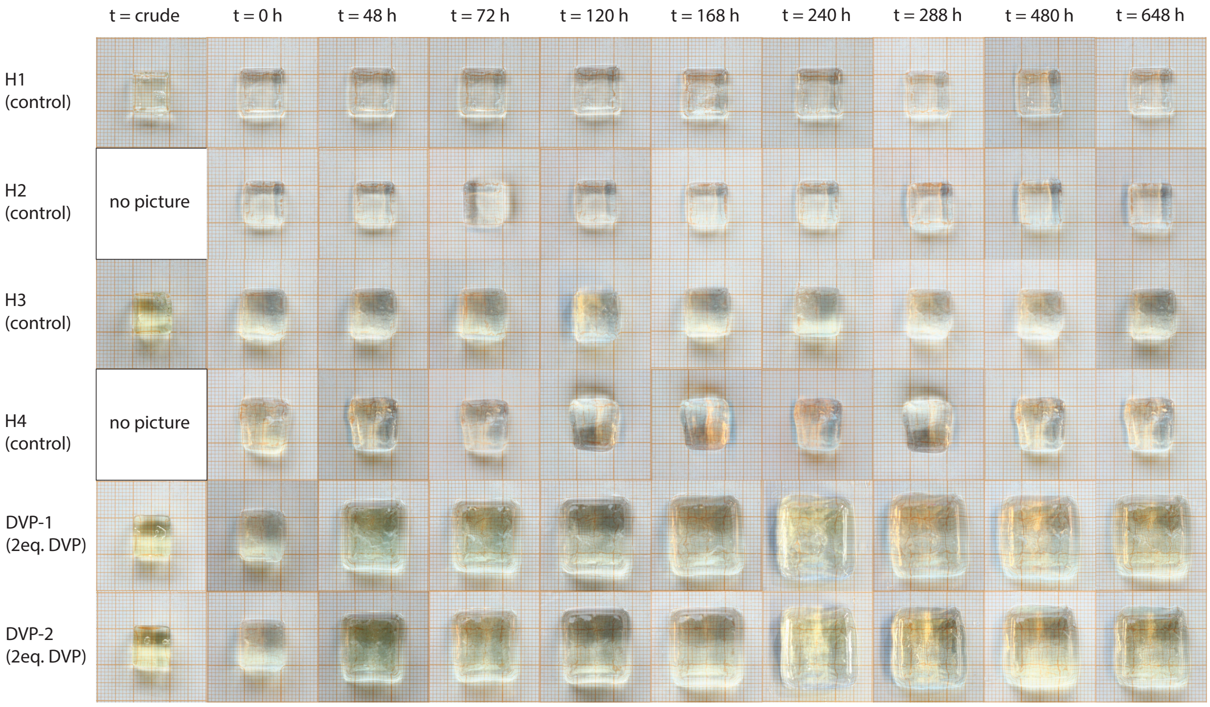

Supplementary Figure 17: Photographs of hydrogels (H1 – H4, DVP 1-2) – crude hydrogels (out of the cast) and equilibrium swollen hydrogel state after t = 96 hours (t = 0). H1-H4 hydrogels were kept blank (no added reactants) and observed via photographs at different time points. DVP-1&2 (duplicate experiment) hydrogels were treated with DVP (2.0 eq.) at t = 0 and observed over time.

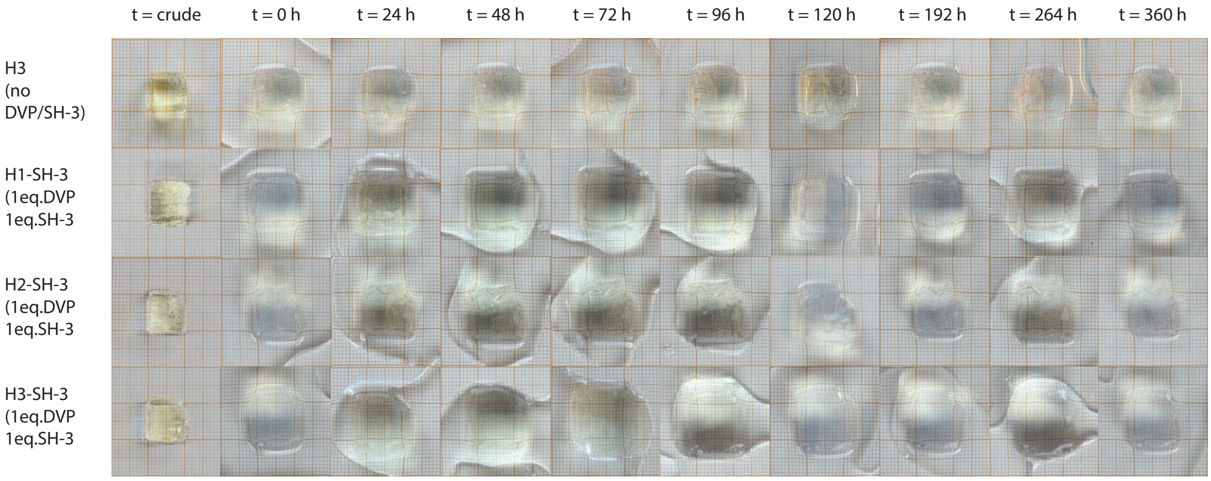

Supplementary Figure 18: Photographs of hydrogels (H3, H1-SH-3 – H3-SH-3) - crude hydrogels (out of the cast) and equilibrium swollen hydrogel state after t = 96 hours in solution (t = 0). H3 hydrogel was kept blank (no added reactants) and observed via photographs at different time points. H1-SH-3 – H3-SH-3 (triplet experiment) hydrogels were treated with DVP (1.0 eq.) at t = 0 and observed over time in solution. At t = 96 hours, SH-3 (1.0 eq.) was added to the hydrogels. After de-swelling at t = 192 hours additional DVP (1.0 eq.) was added to the hydrogels. Then, after re-swelling at t = 264 hours, SH-3 (1.0 eq.) was added to de-swell the hydrogels with full de-swelling at t = 360 hours.

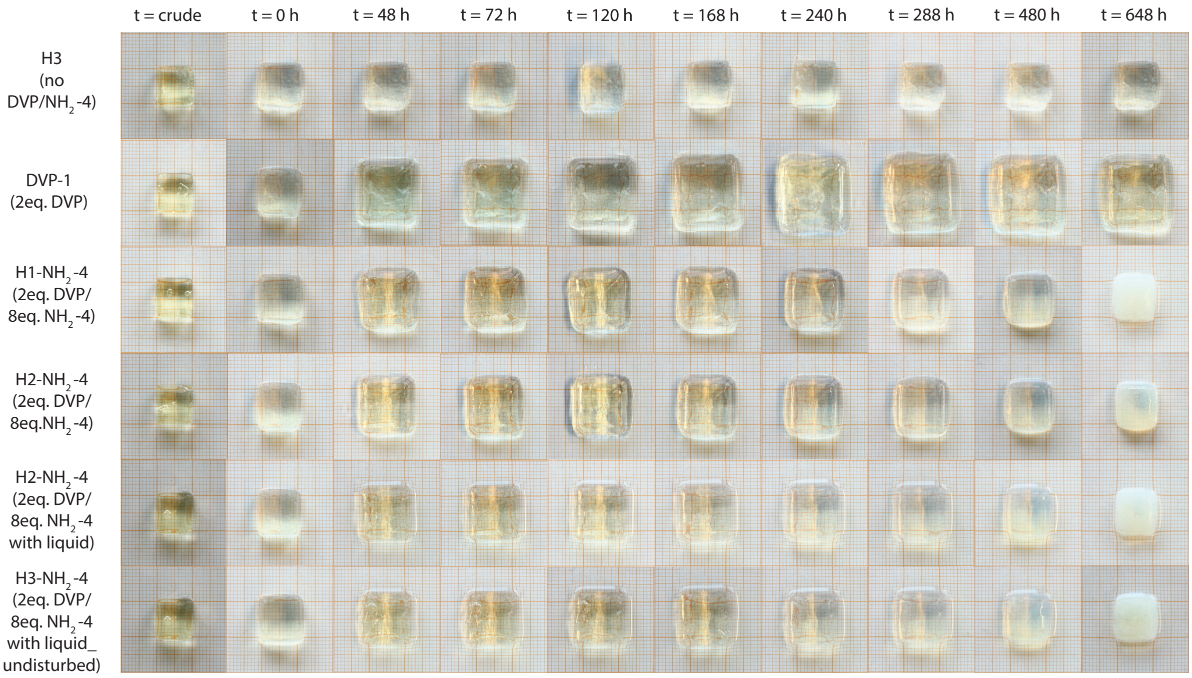

Supplementary Figure 19: Photographs of hydrogels (H3, DVP-1 and H1 to H3-NH_2-4) - crude hydrogels (out of the cast) and equilibrium swollen hydrogel state after t = 96 hours (t = 0). H1 to H3-NH_2-4 hydrogels (triplet experiments) were treated with DVP (2.0 eq.) and NH_2-4 (8.0 eq.) at t = 0 and observed via photographs (no solution) at different time points. H3-NH_2-4 = photographs are with solution (undisturbed sample).

5.0 Synthetic procedures

Synthesis of p(4VP$_{58}$-b-DMA$_{261}$) – polymer for micelles (P1)

P1 was synthesised by a two-step RAFT polymerisation procedure (Scheme 1).

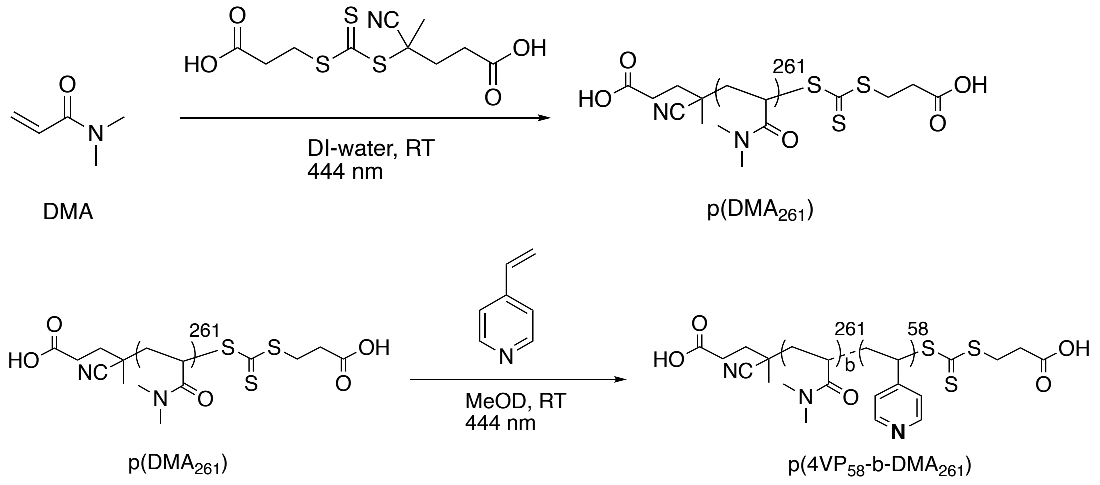

Supplementary Figure 20: Synthetic pathway for the preparation of P1 - p(4VP$_{58}$-b-DMA$_{261}$).

First, the water-soluble block (pDMA$_{261}$) was synthesised as follows. CETCPA (82.2 mg, 0.27 mmol), DMA (7.93 g, 80 mmol), DSS (21.3 mg, 0.10 mmol) and DI water (11.7 mL) were combined in a glass tube sealed with rubber septum. The reaction mixture was deoxygenated by bubbling with argon for 30 minutes and placed into a LED reactor (444 nm). The reaction was quenched after 2 hours (87% conversion by ^{1}H NMR spectroscopy, $M_{n,conv}$ = 26.2 kDa) by removing the glass tube from the light source and opening to air. The polymer was then purified by dialysis using Spectra/por cellulose ester tubing (MWCO 500 – 1000 Da), followed by freeze drying to obtain a light-yellow powder. p(4VP$_{58}$-b) was then obtained by chain-extending pDMA$_{261}$ as follows. pDMA$_{261}$ (655 mg, 0.025 mmol), 4VP (210 mg, 2.0 mmol), DSS (2.0 mg, 0.01 mmol) and MeOD (1.0 mL) were combined and deoxygenated by bubbling with argon for 15 minutes. The solution was then injected into a degassed NMR tube sealed with a rubber septum and placed into the LED reactor (444 nm). The reaction was quenched after 17.5 h irradiation (72% conversion of 4VP by ^{1}H NMR spectroscopy) by removing the glass tube from the light source and opening to air. The polymer was then diluted with ethanol and twice precipitated into diethyl ether (125 mL).

Supplementary Table 2. Polyamine block-copolymer synthesis and characterization data.

Polymer	CTA	[CTA]$_0$:<br>[DMA]$_0$:<br>[4VP]$_0$	Reaction time (h)	NMR conv. (%)	Structure (code)	$M_{n,conv.}$ (kDa)
pDMA$_{261}$	CETCPA	1:300:0	2.0	87 (DMA)	pDMA$_{261}$	26.2
P1	pDMA$_{261}$	1:0:80	18.0	72 (4VP)	p(4VP$_{58}$-b-DMA$_{261}$)	32.3

Supplementary Table 3. Polyamine block-copolymer GPC data.

Polymer	$M_{n,conv}$ (kDa)	$M_{n,GPC}$ (kDa)	$Đ$
$pDMA_{261}$	26.2	28.5	1.14
P1	32.3	29.7	1.23

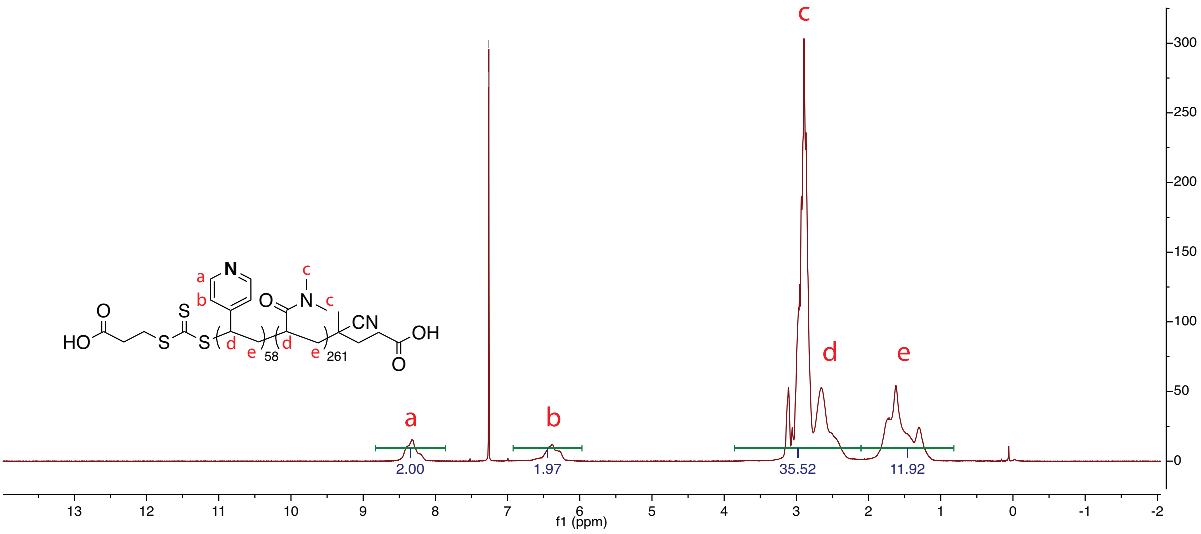

Supplementary Figure 21: ^1H NMR ($CDCl_3$) of P1 demonstrating good (±10%) agreement between polymer structure determined by ^1H NMR conversion and ratio of p(4VP) aromatic signals to polymer backbone. $\int c+d_{th} = [6 \cdot (261) + 1 \cdot (58 + 261)] / 58 = 32.5$; $\int e_{th} = (2) \cdot (261 + 58) / 58 = 11.0$.

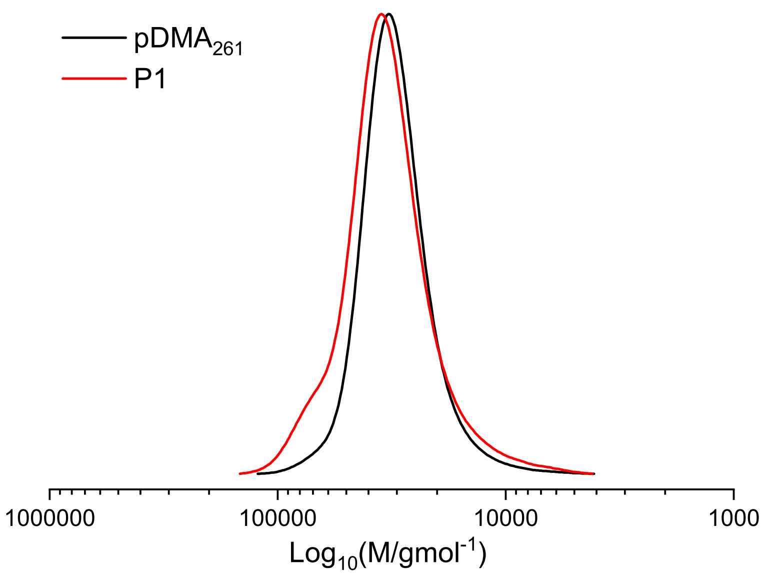

Supplementary Figure 22: GPC traces of $pDMA_{261}$ and chain extended polyamine copolymer P1.

Synthesis of p(4VP$_{28}$-stat-DMA$_{55}$) – P2 precursor used for hydrogel preparation

p(4VP$_{28}$-stat) was synthesised by copolymerising 4VP and DMA, according to Scheme 2.

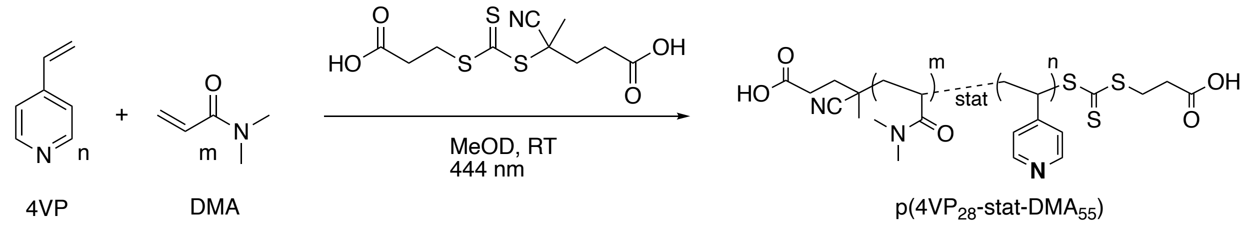

Supplementary Figure 23: Synthetic pathway for the preparation of p(4VP$_{28}$-stat).

CETCPA (82 mg, 0.27 mmol), 4VP (820 mg, 7.80 mmol), DMA (2.40 g, 24.2 mmol) and DMSO (4.5 mL) were combined, chilled in an ice bath, and deoxygenated by bubbling with argon for 30 minutes. The solution was then irradiated in the LED reactor (444 nm) for 45 h, reaching 96% conversion of 4VP and 61% conversion of DMA by ^{1}H NMR spectroscopy. The polymer was isolated by twice precipitation into diethyl ether followed by drying in a vacuum oven.

Supplementary Table 4. Polyamine copolymer synthesis and characterization data.

Polymer	CTA	[CTA]$_0$: [DMA]$_0$: [4VP]$_0$	Reaction time (h)	NMR conv. (%)	Structure	$M_{n,conv.}$ (kDa)
p(4VP$_{28}$-stat)	CETCPA	1:90:30	45.0	61 (DMA) 96 (4VP)	p(4VP$_{28}$-stat-DMA$_{55}$)	8.8

Supplementary Table 5. Polyamine block-copolymer GPC data.

Polymer	$M_{n,conv}$ (kDa)	$M_{n,GPC}$ (kDa)	$Đ$
p(4VP$_{28}$-stat)	8.8	6.3	1.31

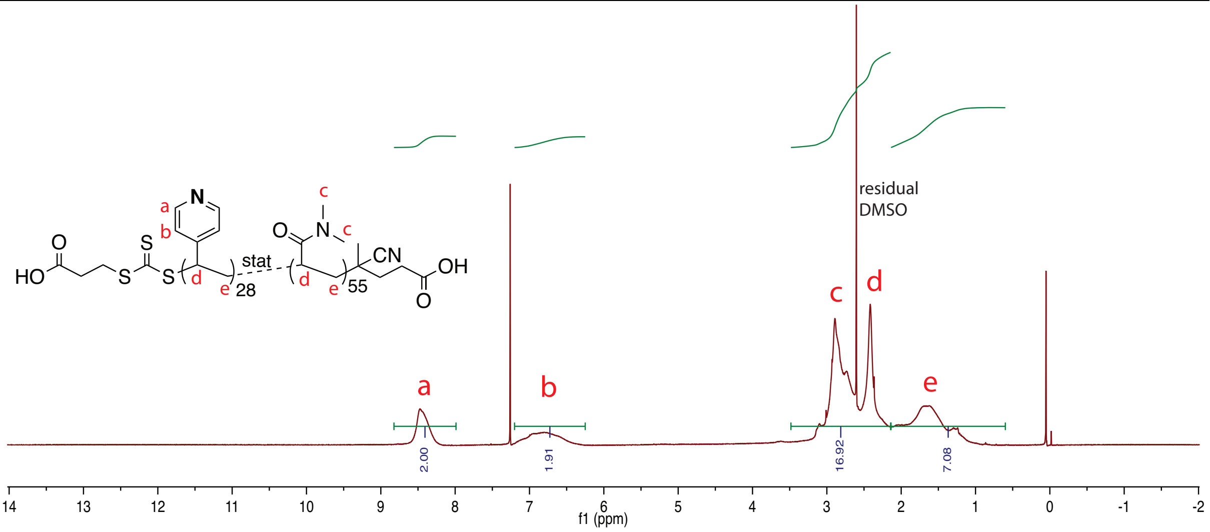

Supplementary Figure 24: ^{1}H NMR (CDCl$_3$) of p(4VP$_{28}$-stat) demonstrating good (±10%) agreement between polymer structure determined by ^{1}H NMR conversion and ratio of p(4VP) aromatic signals to polymer backbone. $\int c+d_{th} = [6 \cdot (55) + 1 \cdot (28 + 55)] / 28 = 14.75$; $\int e_{th} = [2 \cdot (55 + 28) / 28 = 5.9$.

Synthesis of diethyl(α-acetoxymethyl) vinylphosphonate (DVP)

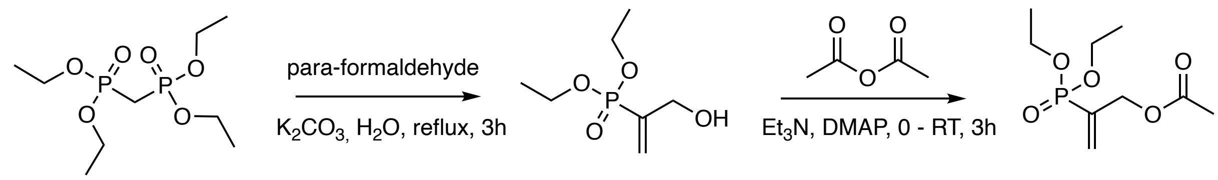

TMP HYP DVP

Supplementary Figure 25: Synthetic pathway for the preparation of DVP.

DVP is a known compound and was synthesized following reported procedures[1,2] (Scheme 3). Briefly, a mixture of tetraethyl methylene diphosphonate (TMP, 20.0 g, 69.4 mmol) in aqueous para-formaldehyde (30%, 50 mL) was heated under reflux. A solution of potassium carbonate (19.2 g, 138.8 mmol in 30 mL of H_2O) was slowly added over a period of 3 hours via a syringe pump. After cooling to room temperature, the reaction mixture was extracted with chloroform (5x 50 mL), washed with brine (2x 50 mL) and dried with Na_2SO_4. Then, the reaction mixture was concentrated under reduced pressure and the residue was subjected to vacuum distillation to afford diethyl (3-hydroxy-2-propenyl)phosphonate (HYP, 10.1 g, 75%) bp: 95 – 105 °C (0.001 torr) as a colourless oil.

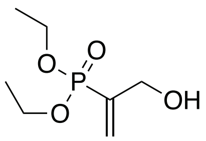

^{1}H NMR (400 MHz, $CDCl_3$) δ: 6.09 (d, J = 2.7 Hz, 1H), 6.01 (d, J = 20.0 Hz, 1H), 4.30 (d, J = 10.7 Hz, 2H), 4.20 – 4.00 (m, 4H), 2.30 (s, 1H), 1.33 (t, J = 7.0 Hz, 6H). **^{13}C NMR** (100.5 MHz, $CDCl_3$) δ: 140.0, 138.3, 128.9 (d, J = 7.2 Hz), 62.6 (d, J = 16.0 Hz), 62.3, 16.4 (d, J = 6.4 Hz). **^{31}P NMR** (161.9 MHz, $CDCl_3$) δ: 17.6 ppm. **MS** (ESI+) m/z: 195.06 (M+H) (expected m/z: 195.07).

To a cooled (0°C) solution of this phosphonate (6.0 g, 30.1 mmol), DMAP (0.227 g, 1.85 mmol) and Et_3N (6.46 mL, 46.35 mmol, 1.5 eq.) in dichloromethane (10 mL) was added dropwise a solution of acetic anhydride (3.21 mL, 34.0 mmol) in dichloromethane (100 mL) and the mixture was stirred at room temperature for 3 h. The reaction mixture was then washed with 15% Na_2CO_3 (2x 100 mL) until pH 9, with 5% HCl (pH 2) and brine before it was dried with Na_2SO_4. Then, the reaction mixture was concentrated under reduced pressure and the residue was purified by flash chromatography (ethyl acetate/methanol, 95:5) to furnish diethyl(α-acetoxymethyl) vinylphosphonate as a colourless oil (DVP, 6.1 g, 84%).

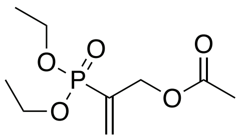

^{1}H NMR (400 MHz, $CDCl_3$) δ: 6.19 (dt, J = 22.4, 1.4 Hz, 1H), 6.01 (dt, J = 45.8, 1.6 Hz, 1H), 4.72 (dt, J = 8.9, 1.5 Hz, 2H), 4.23 – 3.93 (m, 4H), 2.09 (d, J = 1.3 Hz, 3H), 1.32 (t, J = 7.1, 1.3 Hz, 6H). **^{13}C NMR** (100.5 MHz, $CDCl_3$) δ: 170.3, 135.6, 133.8, 131.1 (d, J = 7.0 Hz), 63.1, 62.9, 62.3 (d, J = 5.7 Hz), 20.9, 16.4 (d, J = 6.3 Hz). **^{31}P NMR** (161.9 MHz, $CDCl_3$) δ: 15.9 ppm. **MS** (ESI+) m/z: 237.07 (M+H) (expected m/z: 237.08).

Synthesis of 1-(2-(diethoxyphosphoryl)allyl)-1,4 diazabicyclo[2.2.2] octanium chloride (DVP-t-Am-1)

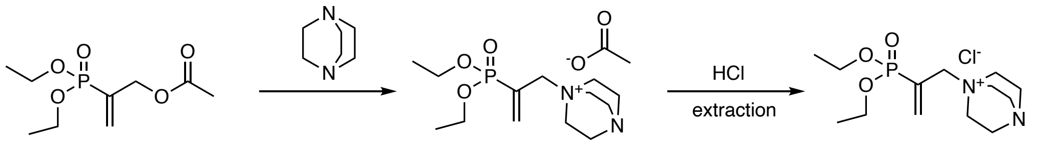

DVP   DVP -t-Am-**1**, not isolated   DVP -t-Am-**1**, isolated

Supplementary Figure 26: Synthetic pathway for the preparation of DVP-t-Am-1.

To a solution of DVP (20 mg, 85 μmol, 1.1 eq.) in 1.0 mL D_2O was added (1,4-diazabicyclo[2.2.2]octane) (t-Am-**1**, 1.0 eq.) and shaken for 10 min. The reaction progress was monitored by NMR. After the completion of the reaction, the mixture was extracted with chloroform. Hereafter, a few drops of 1 M HCl were added and then the mixture was extracted with chloroform (3x, 1 mL) again. The water layer was freeze dried to afford 1-(2-(diethoxyphosphoryl)allyl)-1,4-diazabicyclo[2.2.2]octanium chloride (DVP-t-Am-**1**) as colourless, hydroscopic oil (17 mg, 62%).

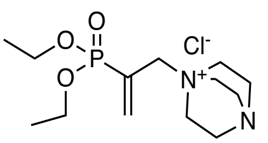

^{1}H NMR (400 MHz, DMSO-d_6) δ: 6.65 (d, J = 20.4 Hz, 1H), 6.57 (d, J = 3.0 Hz, 1H), 4.17 (d, J = 16.0 Hz, 2H), 4.12 – 4.01 (m, 4H), 3.39 (t, J = 7.4 Hz, 6H), 3.05 (t, J = 7.4 Hz, 6H), 1.28 (t, J = 7.0 Hz, 6H). **^{13}C NMR** (100.5 MHz, DMSO-d_6) δ: 145.4 (d, J = 6.8 Hz), 62.9 (d, J = 13.7 Hz), 62.5 (d, J = 6.2 Hz), 52.1, 44.7, 16.1 (d, J = 6.0 Hz). **^{31}P NMR** (161.9 MHz, DMSO-d_6) δ: 15.3 ppm. **MS** (ESI+) m/z: 289.18 (M-Cl) (expected m/z: 289.17).

Synthesis of 1-(2-(diethoxyphosphoryl)allyl)pyridinium chloride (DVP-t-Am-2)

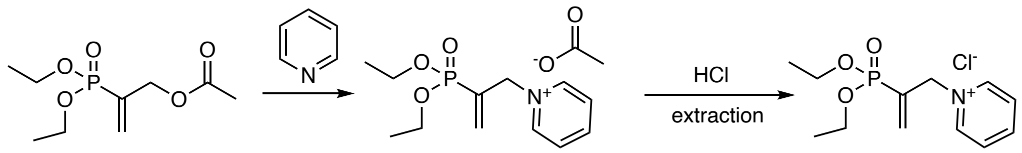

DVP   DVP -t-Am-**2**, not isolated   DVP -t-Am-**2**, isolated

Supplementary Figure 27: Synthetic pathway for the preparation of DVP-t-Am-2.

To a solution of DVP (20 mg, 85 μmol, 1.1 eq.) in 1.0 mL D_2O was added pyridine (t-Am-**2**, 1.0 eq.) and shaken for 10 min. The reaction progress was monitored by NMR. After the completion of the reaction, the product was isolated by extraction with chloroform. Hereafter, few drops of 1 M HCl were added and then the mixture was extracted with chloroform (3x, 1 mL) again. The water layer was freeze dried to afford 1-(2-(diethoxyphosphoryl)allyl)pyridinium chloride (DVP-t-Am-**2**) as colourless, hydroscopic oil (16 mg, 65%).

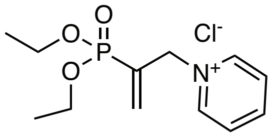

^{1}H NMR (400 MHz, DMSO-d_6) δ: 9.15 (d, *J* = 5.2 Hz, 2H), 8.69 (t, *J* = 7.8 Hz, 1H), 8.35 – 8.09 (m, 2H), 6.45 (d, *J* = 44.0 Hz, 1H), 6.30 (d, *J* = 20.9 Hz, 1H), 5.59 (d, *J* = 13.8 Hz, 2H), 3.99 – 3.84 (m, 4H), 1.12 (t, *J* = 7.1 Hz, 6H). **^{13}C NMR** (100.5 MHz, DMSO-d_6) δ: 146.5, 145.4, 137.5 (d, *J* = 7.6 Hz), 133.9, 132.1, 128.0, 62.1 (d, *J* = 5.8 Hz), 61.2 (d, *J* = 12.4 Hz), 15.9 (d, *J* = 6.1 Hz).**^{31}P NMR** (161.9 MHz, DMSO-d_6) δ: 14.4 ppm. **MS** (ESI+) m/z: 256.10 (M-Cl) (expected m/z: 256.11).

Synthesis of 3-(2-hydroxyethylsulfanyl)prop-1-en-2-ylphosphonate (DVP-S)

To a solution of DVP (20 mg, 85 µmol, 1.0 eq.) in D_2O/phosphate buffer 1:9 (0.1 M, pH = 7.4) mixture was added t-Am-**1** (17 µmol, 0.2 eq.) and SH-**3** (93 µmol, 1.1 eq.) and the mixture was stirred at RT overnight. After lyophilization, the crude was purified by flash chromatography (ethyl acetate/methanol, 95:5) to furnish diethyl 3-(2-hydroxyethylsulfanyl)prop-1-en-2-ylphosphonate (DVP-S) as a colourless oil (16 mg, Yield: 74%).

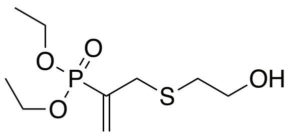

^{1}H NMR (400 MHz, $CDCl_3$) δ: 6.06 (d, *J* = 1.2 Hz, 1H), 6.03 (d, *J* = 67.6 Hz, 0H), 4.26 – 3.98 (m, 4H), 3.74 (t, *J* = 6.5 Hz, 2H), 3.40 (d, *J* = 13.3 Hz, 2H), 2.86 (s, 1H), 2.69 (t, *J* = 5.8 Hz, 2H), 1.33 (t, *J* = 7.1 Hz, 6H). **^{13}C NMR** (100.5 MHz, $CDCl_3$) δ: 137.1, 135.3, 131.3 (d, *J* = 8.8 Hz), 62.4 (d, *J* = 5.7 Hz), 60.6, 35.2, 33.2 (d, *J* = 13.7 Hz), 16.5 (d, *J* = 6.3 Hz). **^{31}P NMR** (161.9 MHz, $CDCl_3$) δ: 17.6 ppm. **MS** (ESI+) m/z: 255.08 (M+H) (expected m/z: 254.07).

Synthesis of 2-((2-(diethoxyphosphoryl)allyl)amino)-3-hydroxybutanoic acid (DVP-N)

To a solution of DVP (20 mg, 85 µmol, 1.0 eq.) in D_2O/phosphate buffer 1:9 (0.1 M, pH = 7.4) mixture was added t-Am-**1** (17 µmol, 0.2 eq.) and NH_2-**4** (85 µmol, 1.0 eq.) and the mixture was stirred at RT for 4 days. After lyophilization, the crude was solubilized in EtOAc and filtered. 2-((2-(diethoxyphosphoryl)allyl)amino)-3-hydroxybutanoic acid (DVP-N) was obtained by recrystallization in EtOAc as a white solid (15 mg, Yield: 60%).

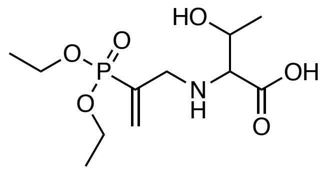

^{1}H NMR (400 MHz, methanol-d_4) δ: 6.39 (d, J = 5.4 Hz, 1H), 6.31 (d, J = 18.6 Hz, 1H), 4.31 – 4.06 (m, 4H), 4.04 – 3.97 (m, 1H), 3.97 – 3.76 (m, 2H), 3.30 (s, 1H), 1.39 (t, J = 3.7 Hz, 6H), 1.37 (t, J = 1.7 Hz, 3H). **^{13}C NMR** (100.5 MHz, methanol-d_4) δ: 171.6, 137.3 (d, J = 7.5 Hz), 132.3 (d, J = 180.8 Hz), 69.8, 67.4, 64.6 (dd, J = 6.2, 3.2 Hz), 21.4, 16.6 (d, J = 5.9 Hz). **^{31}P NMR** (161.9 MHz, MeOD) δ: 16.1. **MS** (ESI+) m/z: 296.14 (M+H) (expected m/z: 296.27).

6.0 NMR Spectra

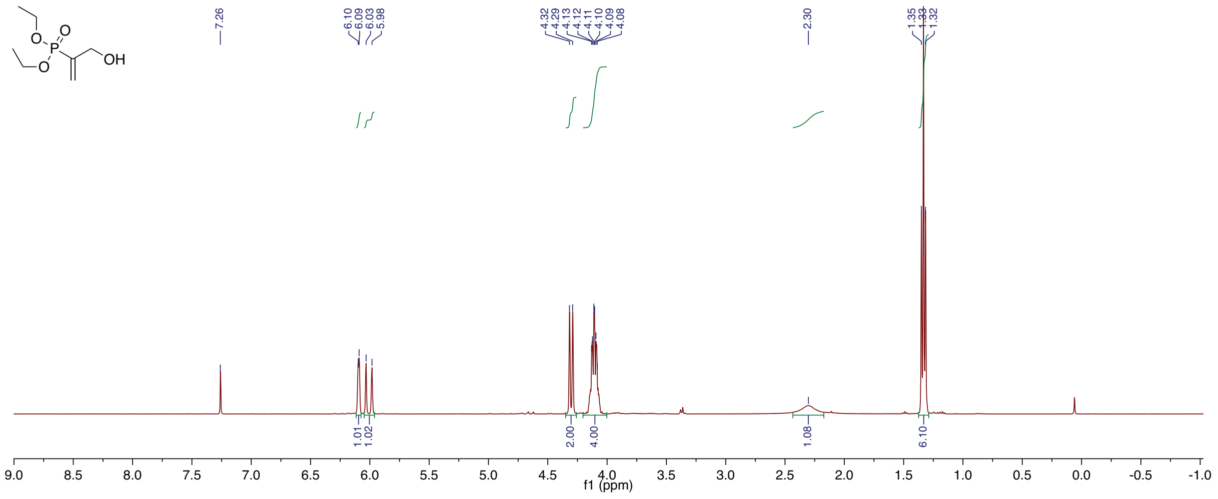

Supplementary Figure 28: ^{1}H NMR, HYP in $CDCl_3$.

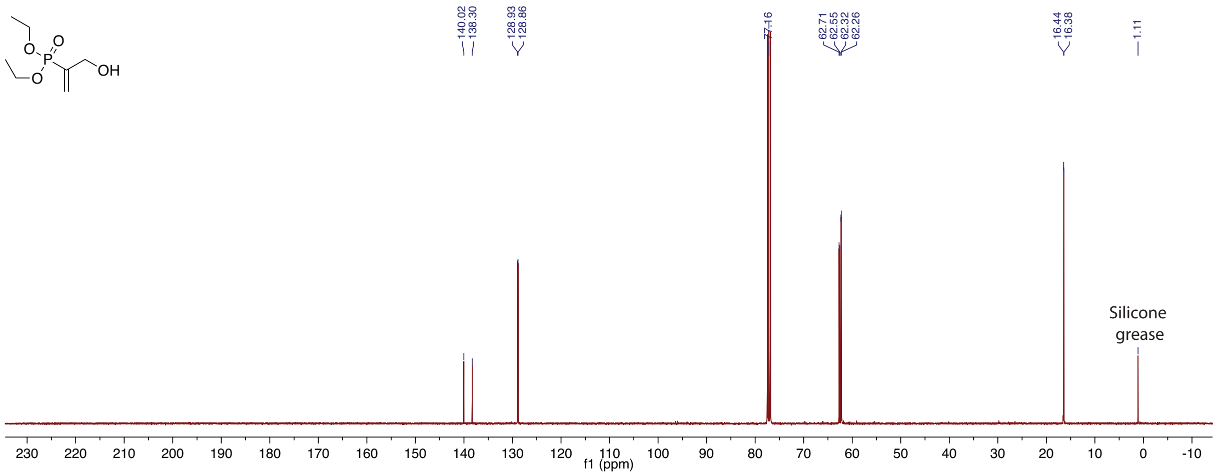

Supplementary Figure 29: ^{13}C NMR, HYP in $CDCl_3$.

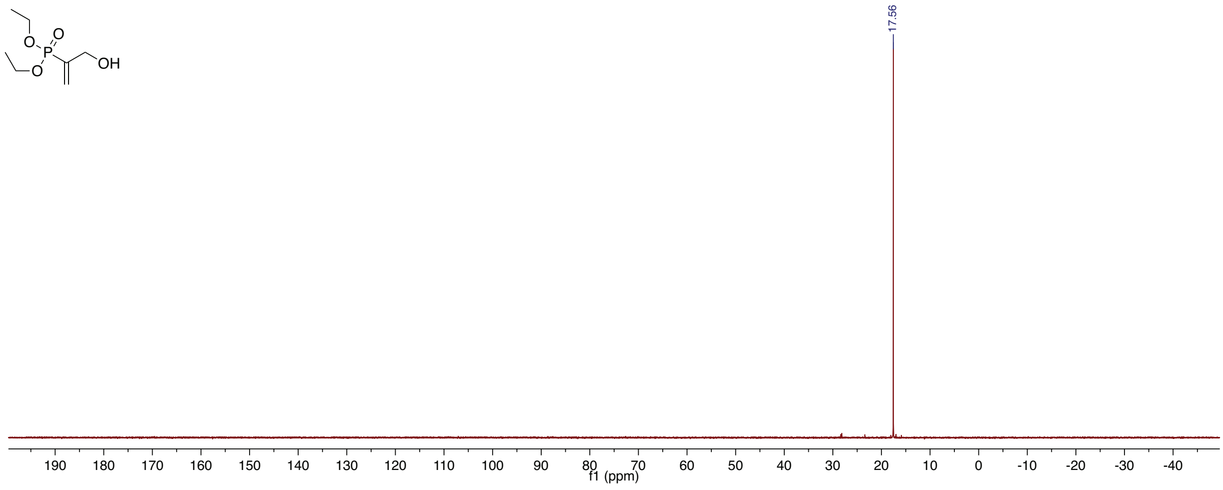

Supplementary Figure 30: ^{31}P NMR, HYP in $CDCl_3$.

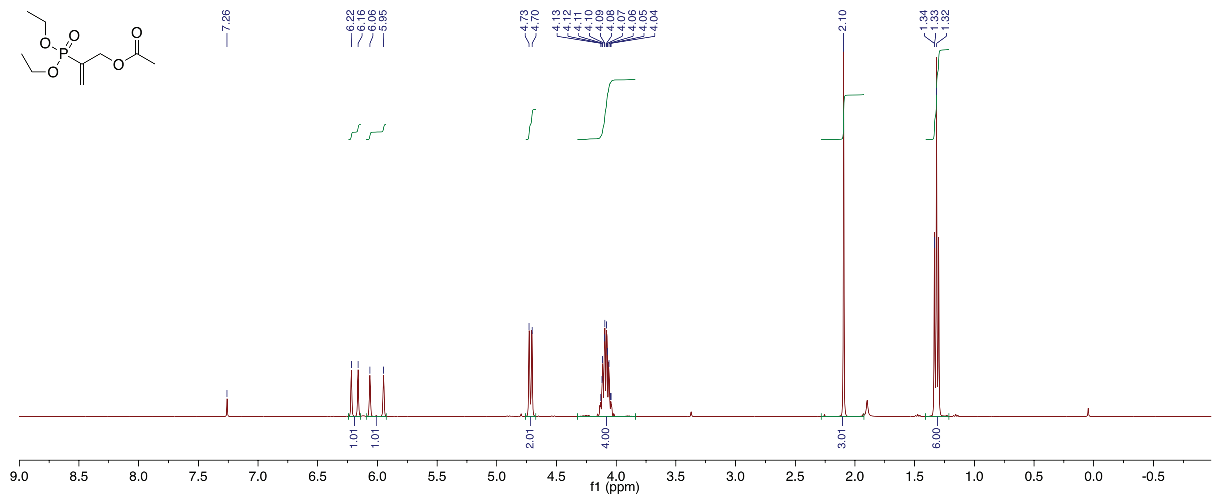

Supplementary Figure 31: ^{1}H NMR, DVP in $CDCl_3$.

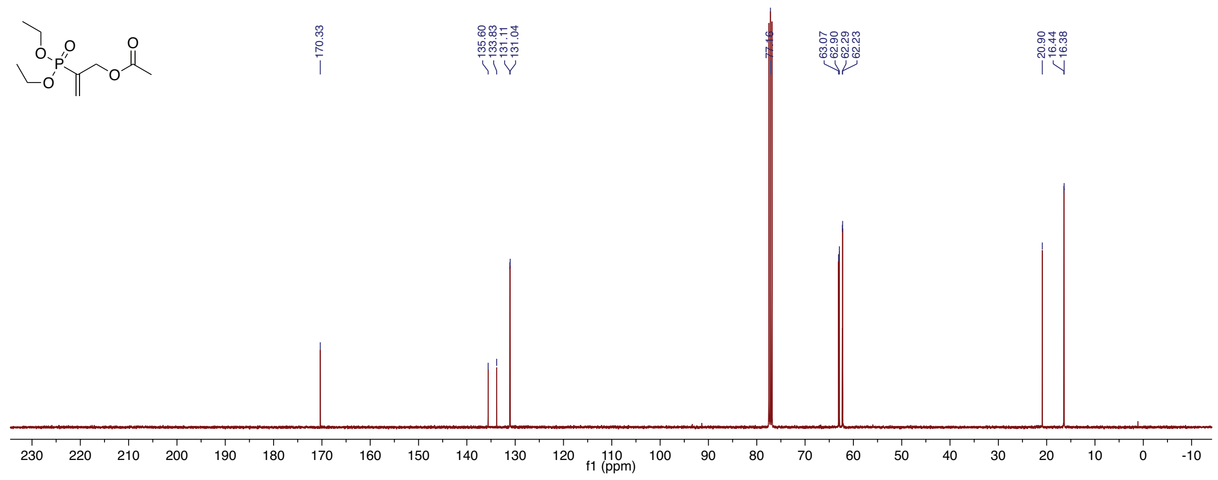

Supplementary Figure 32: ^{13}C NMR, DVP in $CDCl_3$.

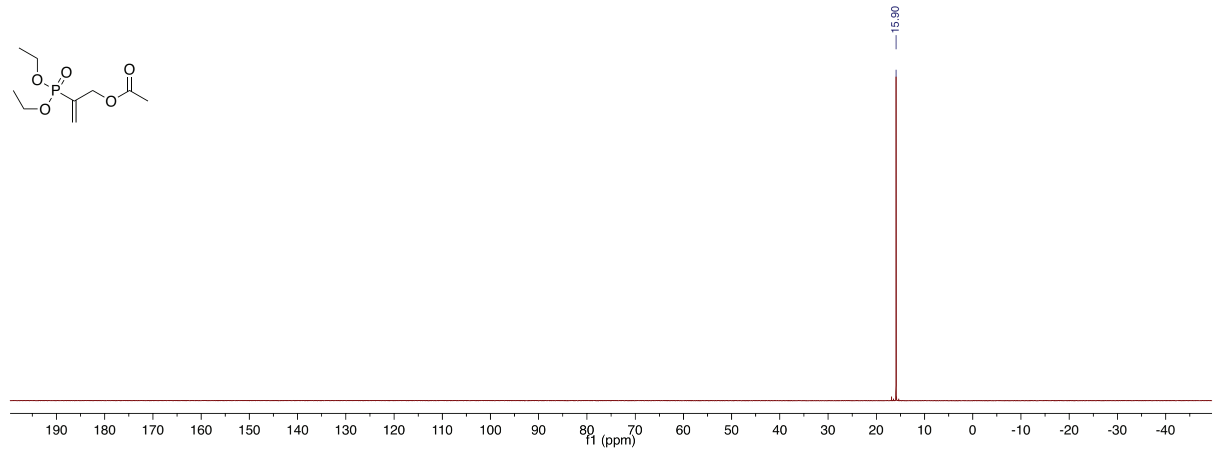

Supplementary Figure 33: ^{31}P NMR, DVP in $CDCl_3$.

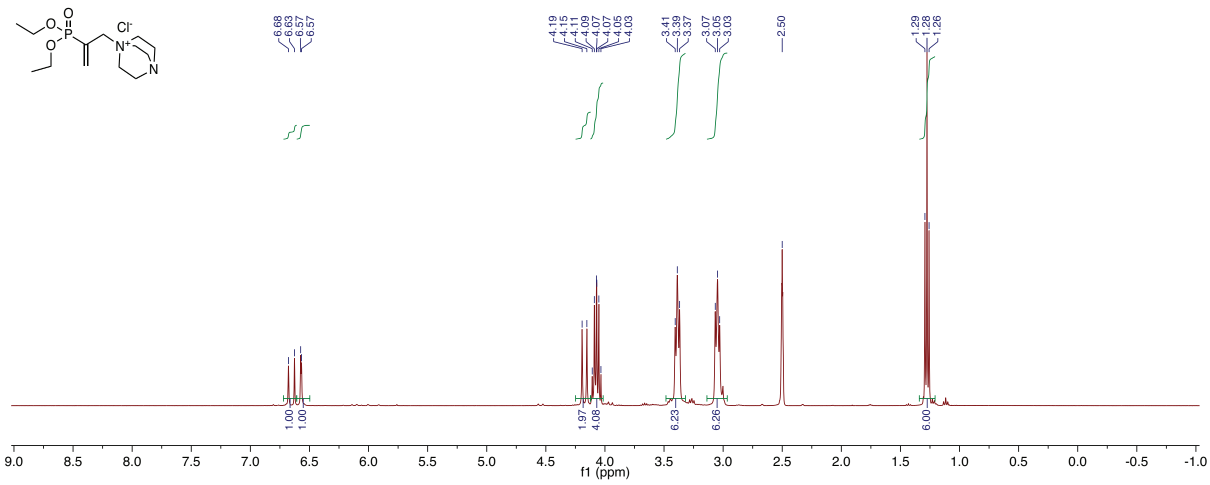

Supplementary Figure 34: ^{1}H NMR, DVP-t-Am-1 in DMSO-d_6.

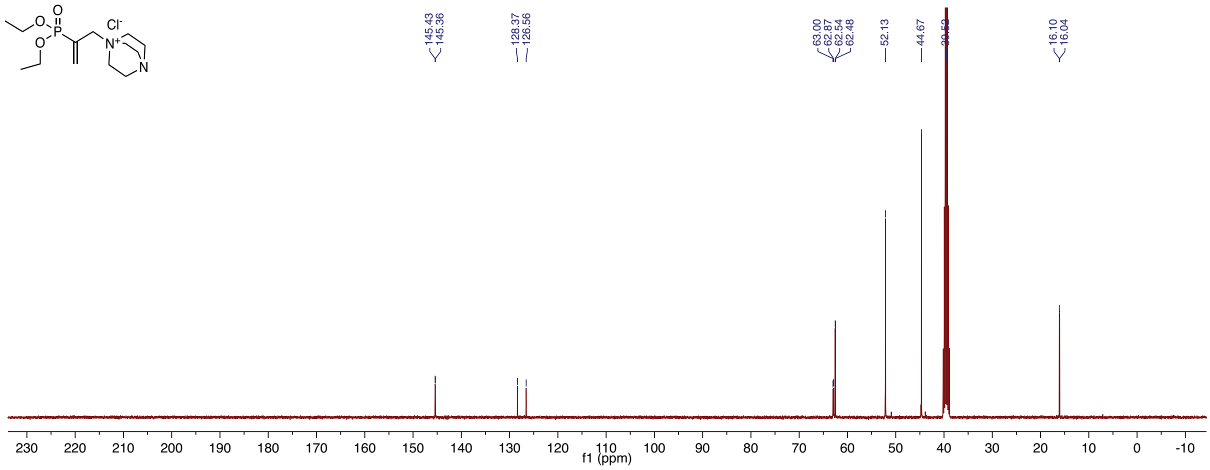

Supplementary Figure 35: ^{13}C NMR, DVP-t-Am-1 in DMSO-d_6.

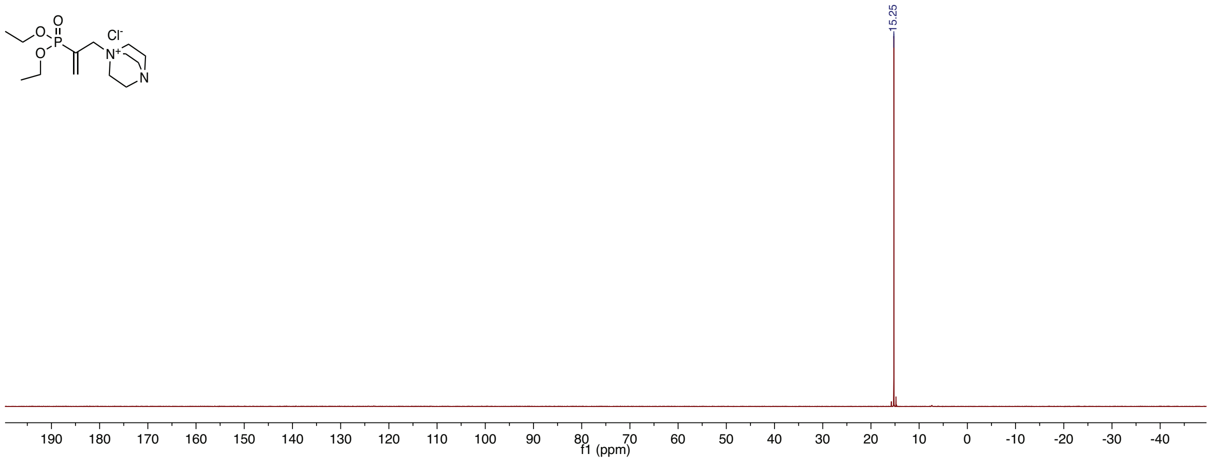

Supplementary Figure 36: ^{31}P NMR, DVP-t-Am-1 in DMSO-d_6.

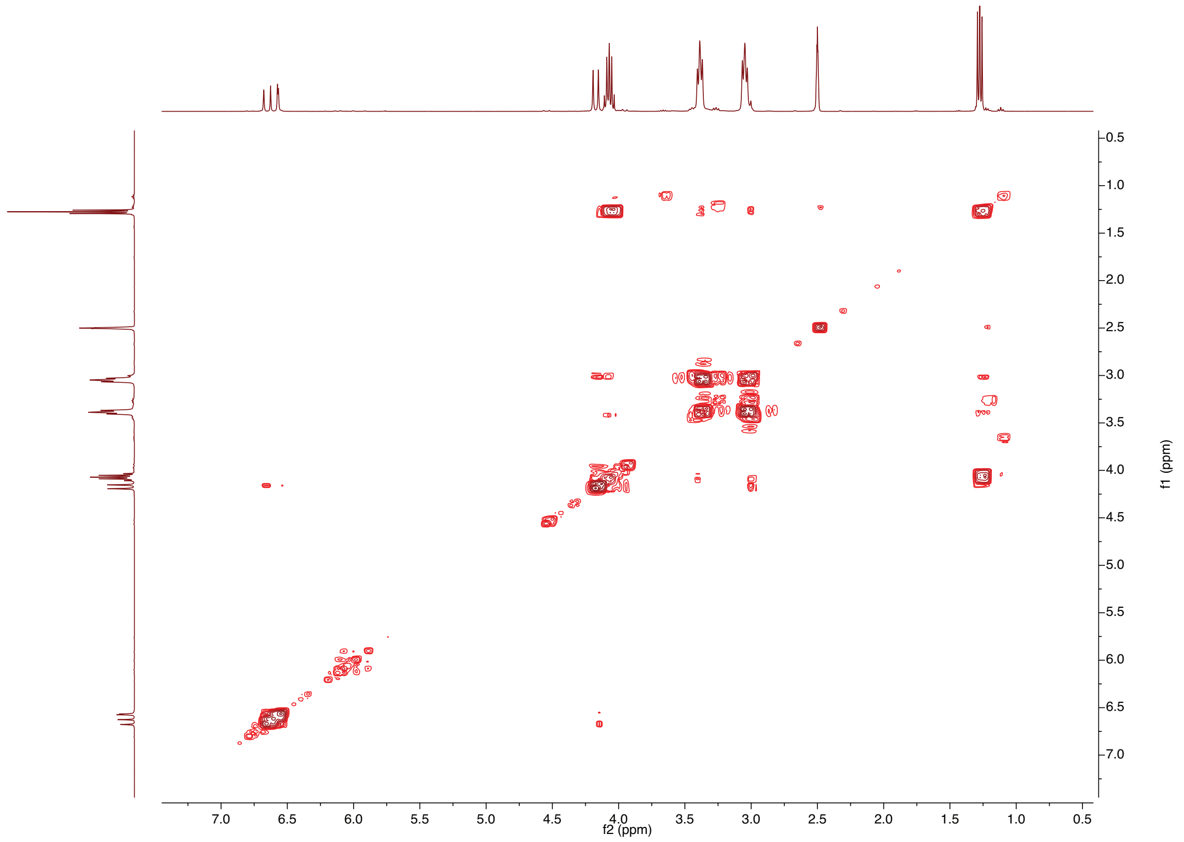

Supplementary Figure 37: gCOSY NMR, DVP-t-Am-1 in DMSO-d_6.

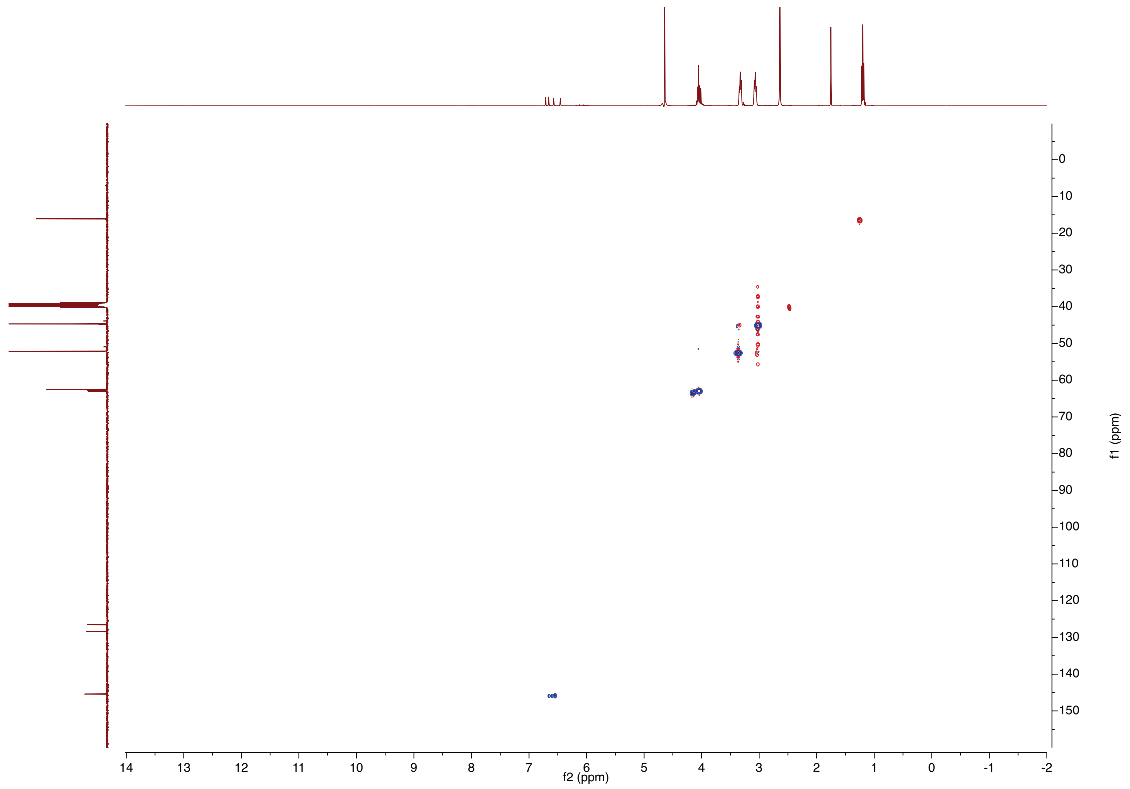

Supplementary Figure 38: gHSQC NMR, DVP-t-Am-1 in DMSO-d_6.

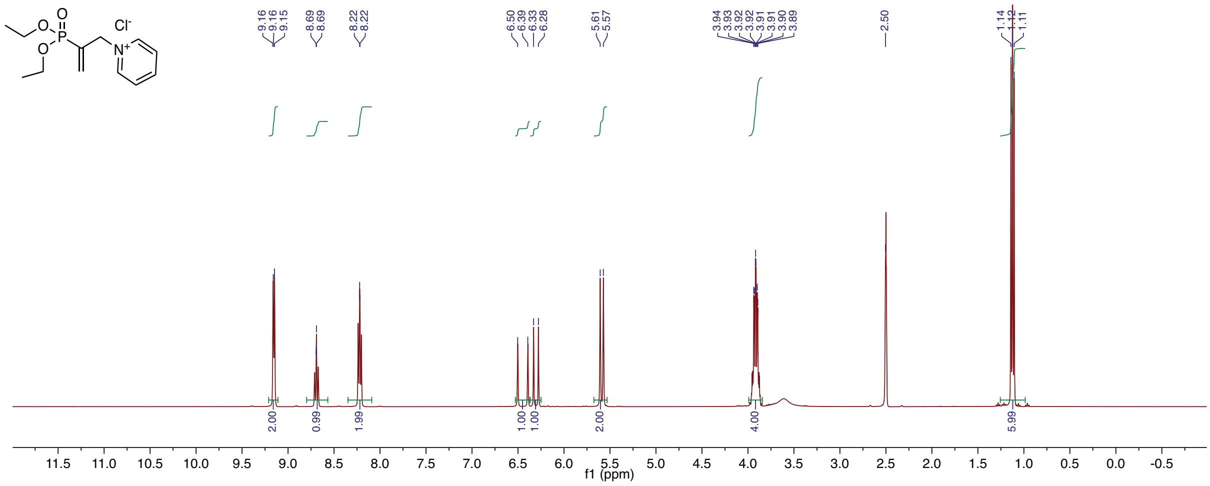

Supplementary Figure 39: ^{1}H NMR, DVP-t-Am-2 in DMSO-d_6.

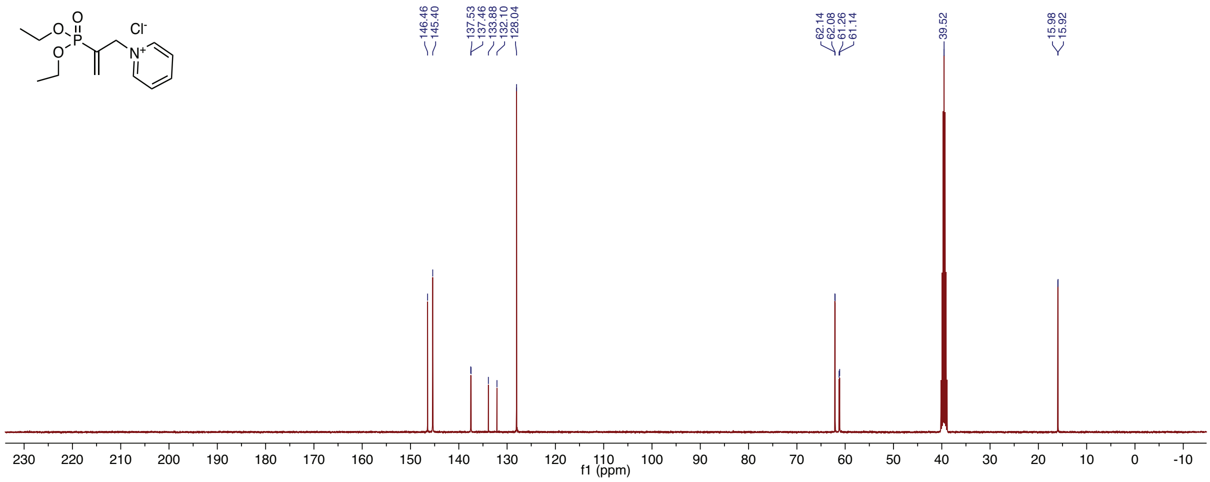

Supplementary Figure 40: ^{13}C NMR, DVP-t-Am-2 in DMSO-d_6.

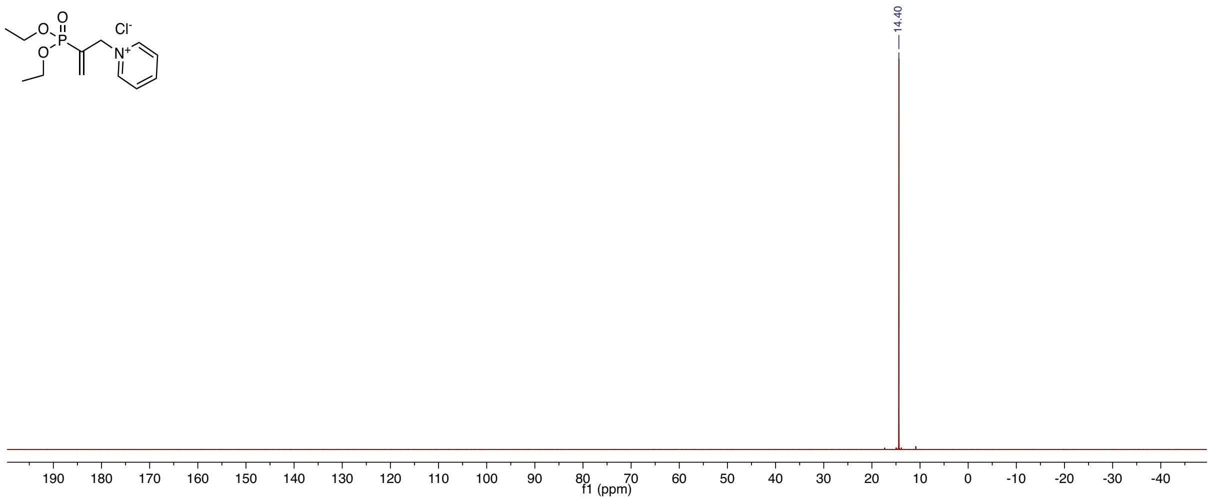

Supplementary Figure 41: ^{31}P NMR, DVP-t-Am-2 in DMSO-d_6.

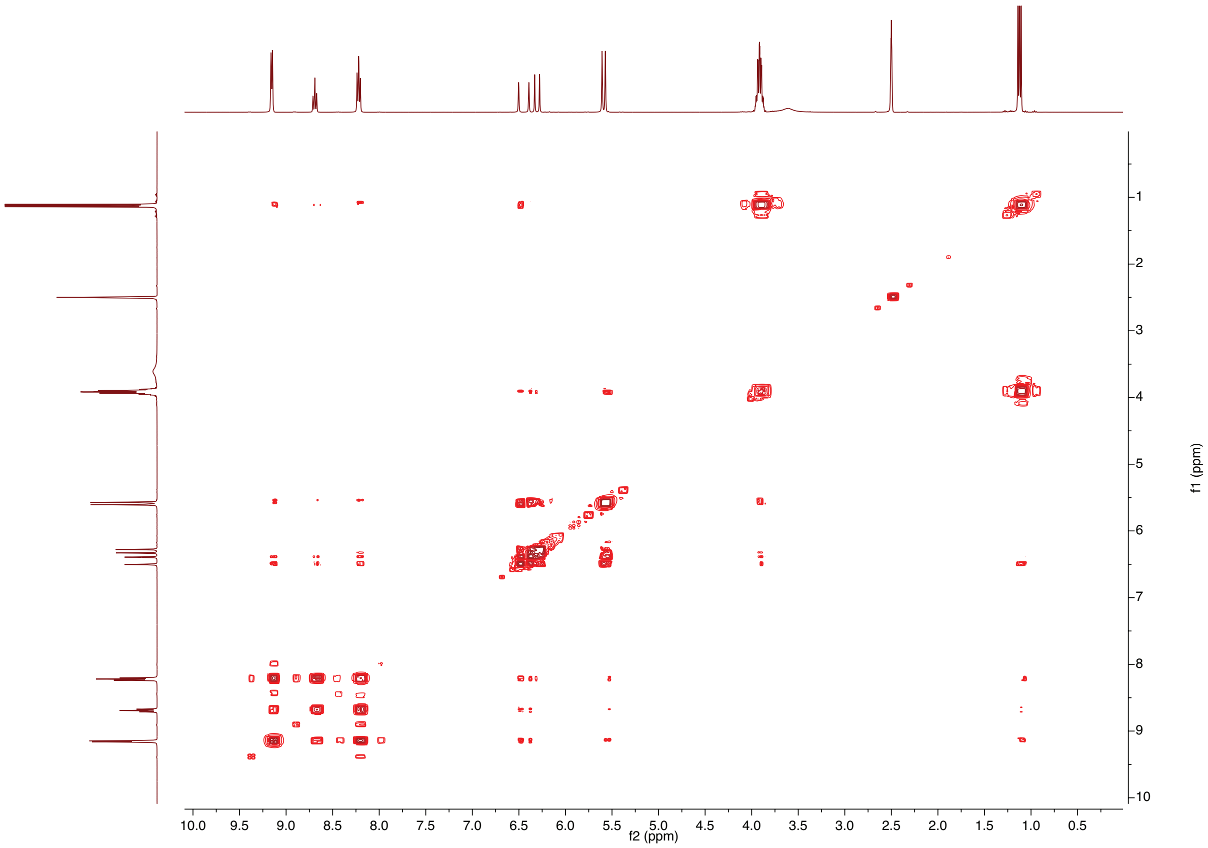

Supplementary Figure 42: gCOSY, DVP-t-Am-2 in DMSO-*d*6.

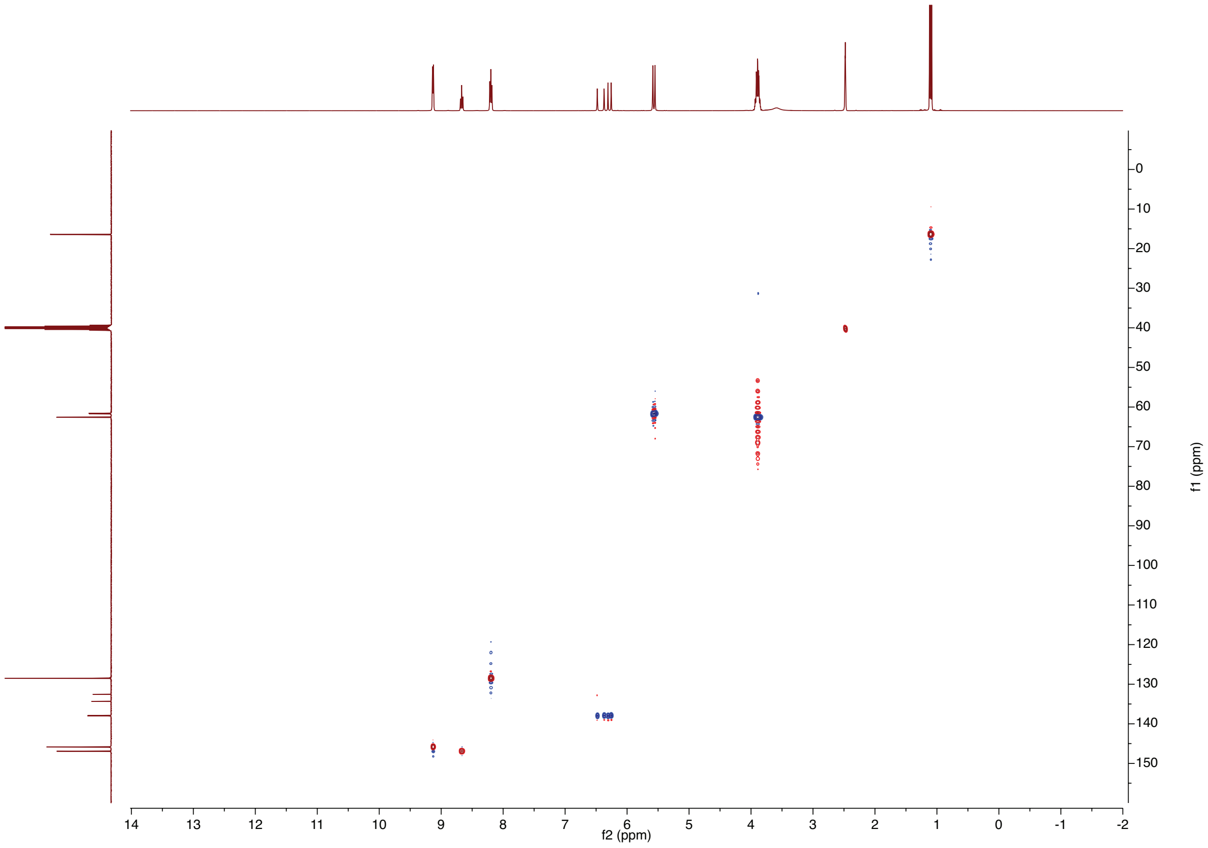

Supplementary Figure 43: gHSQC, DVP-t-Am-2 in DMSO-*d*6.

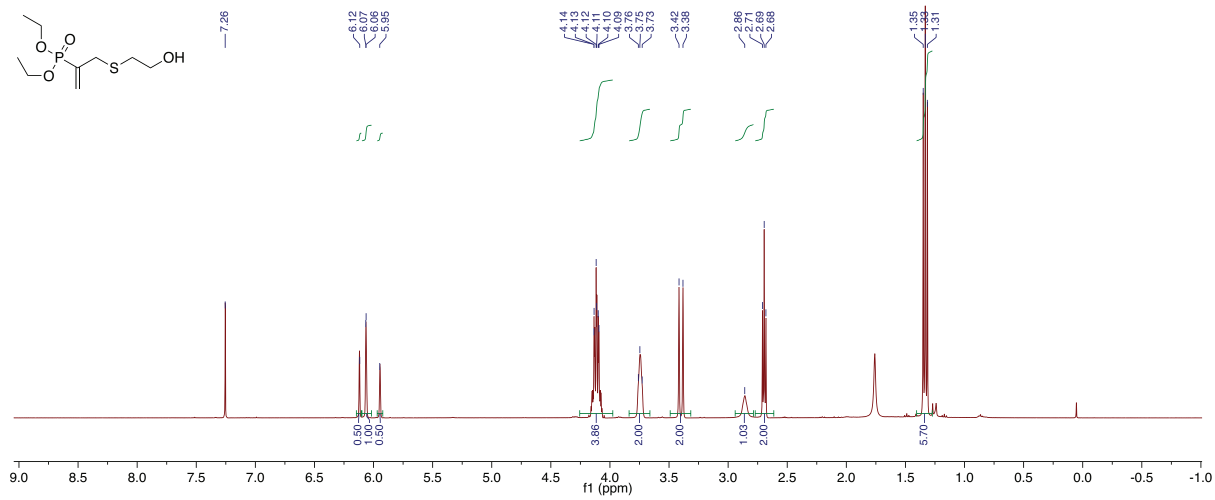

Supplementary Figure 44: ^{1}H NMR, DVP-S in $CDCl_3$.

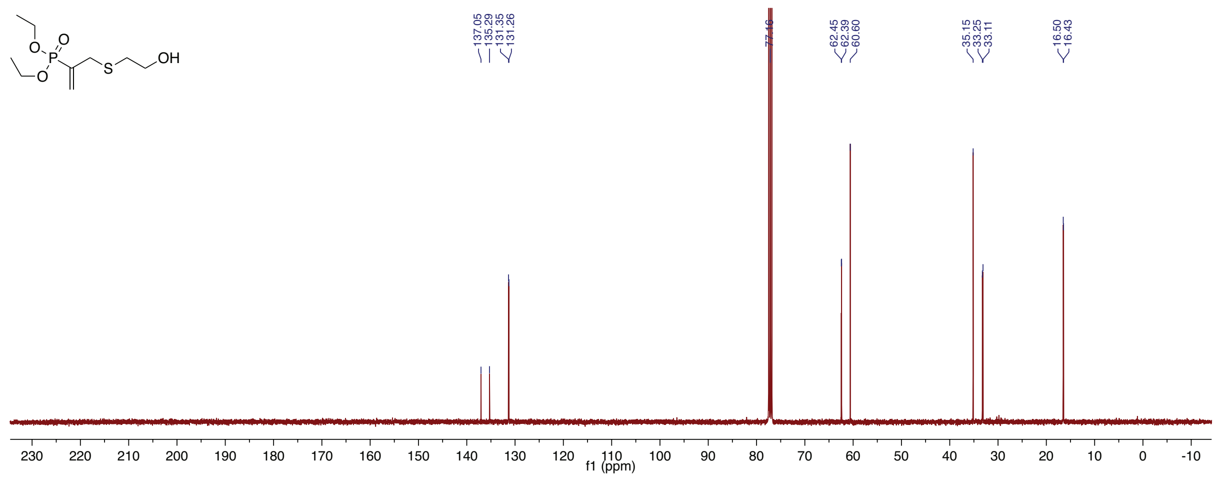

Supplementary Figure 45: ^{13}C NMR, DVP-S in $CDCl_3$.

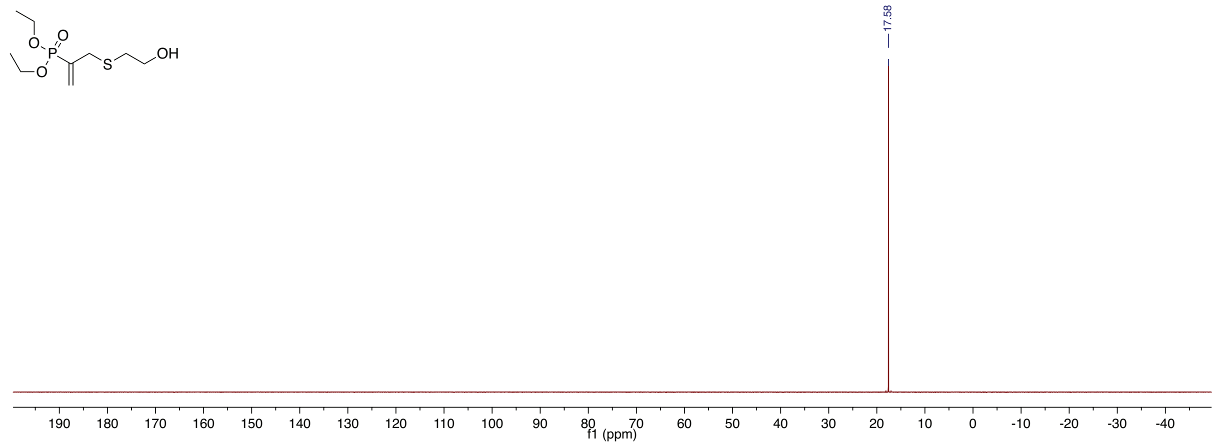

Supplementary Figure 46: ^{31}P NMR, DVP-S in $CDCl_3$.

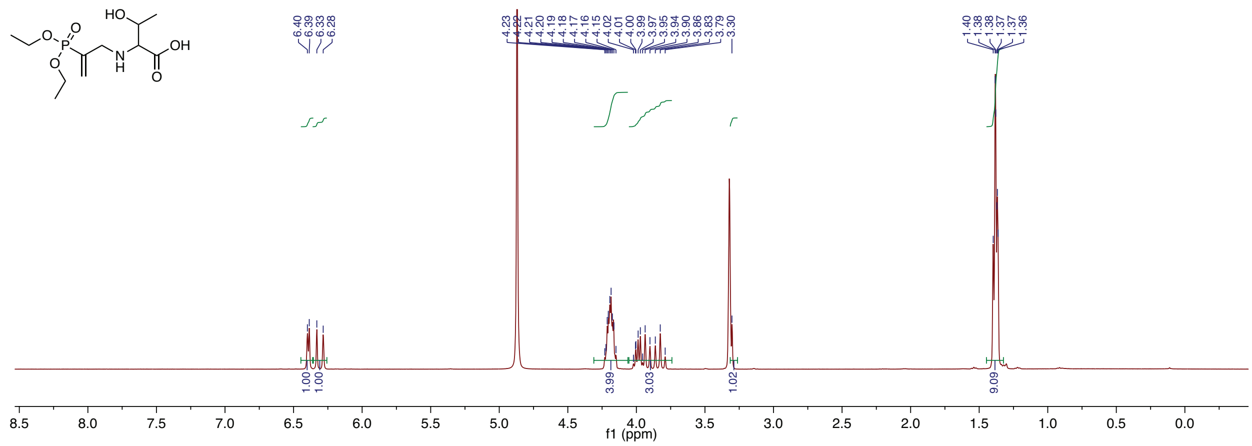

Supplementary Figure 47: ^{1}H NMR, DVP-N in methanol-d_4.

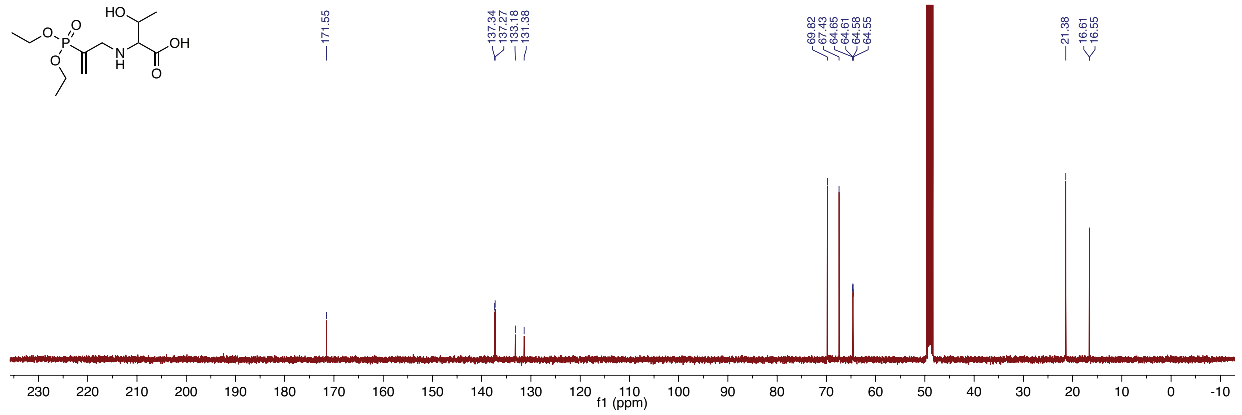

Supplementary Figure 48: ^{13}C NMR, DVP-N in methanol-d_4.

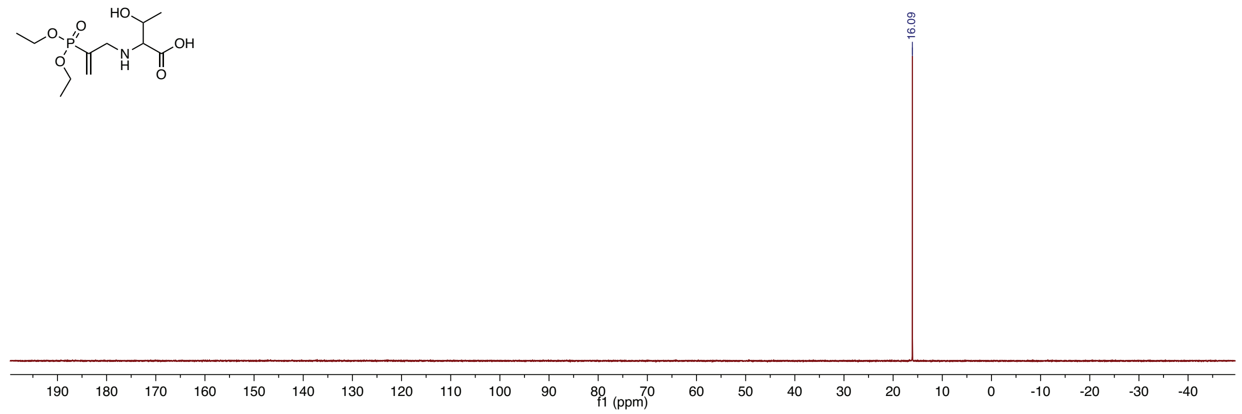

Supplementary Figure 49: ^{31}P NMR, DVP-N in methanol-d_4.

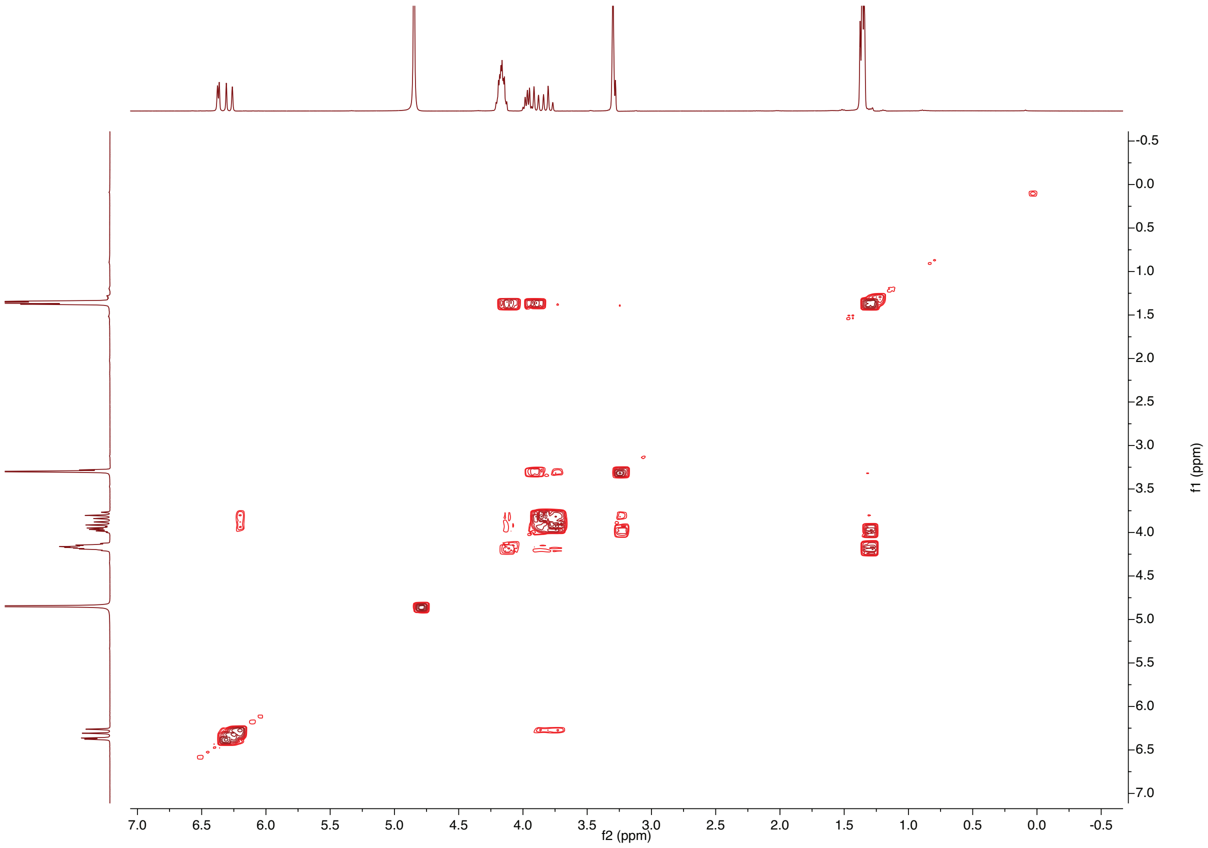

Supplementary Figure 50: gCOSY, DVP-N in methanol-d$_4$.

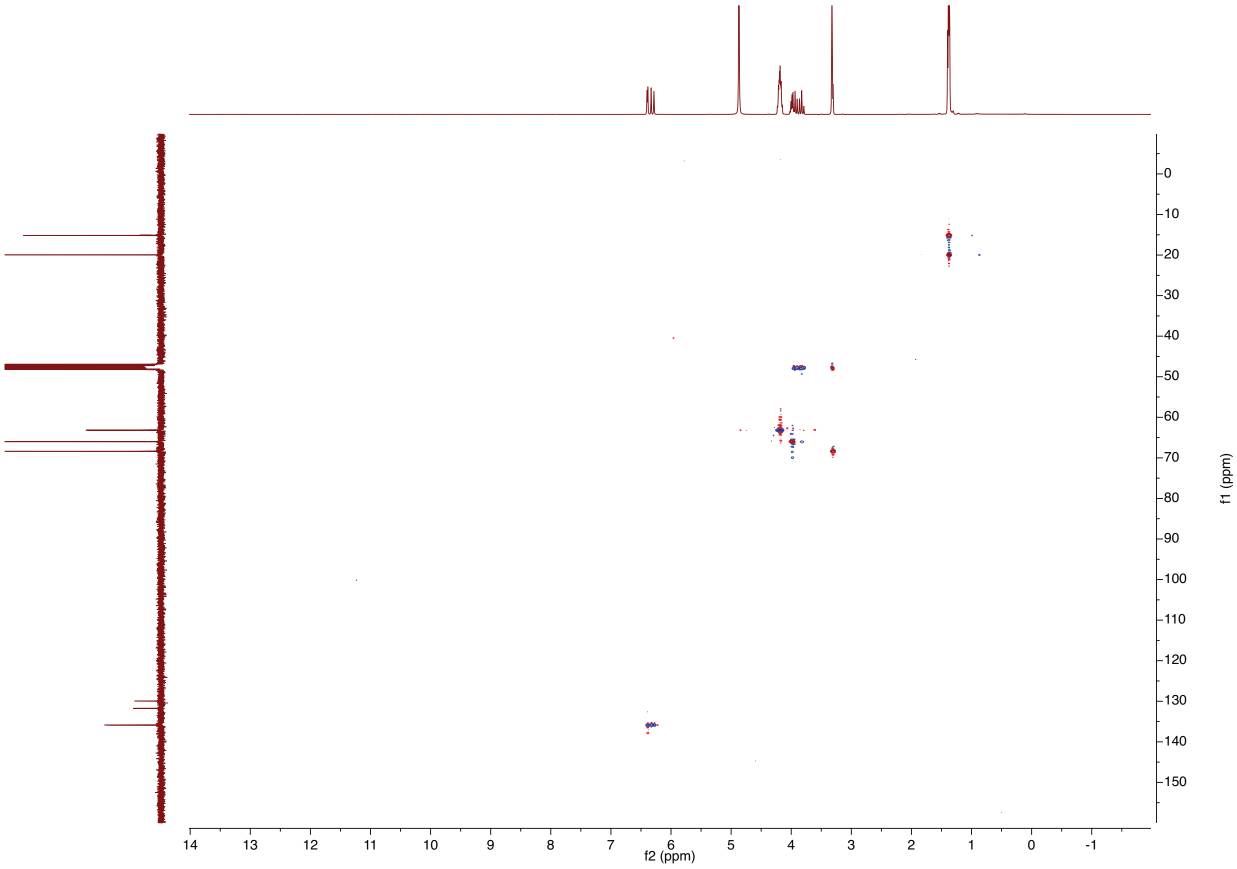

Supplementary Figure 51: gHSQC, DVP-N in methanol-d$_4$.

7.0 LC-MS data

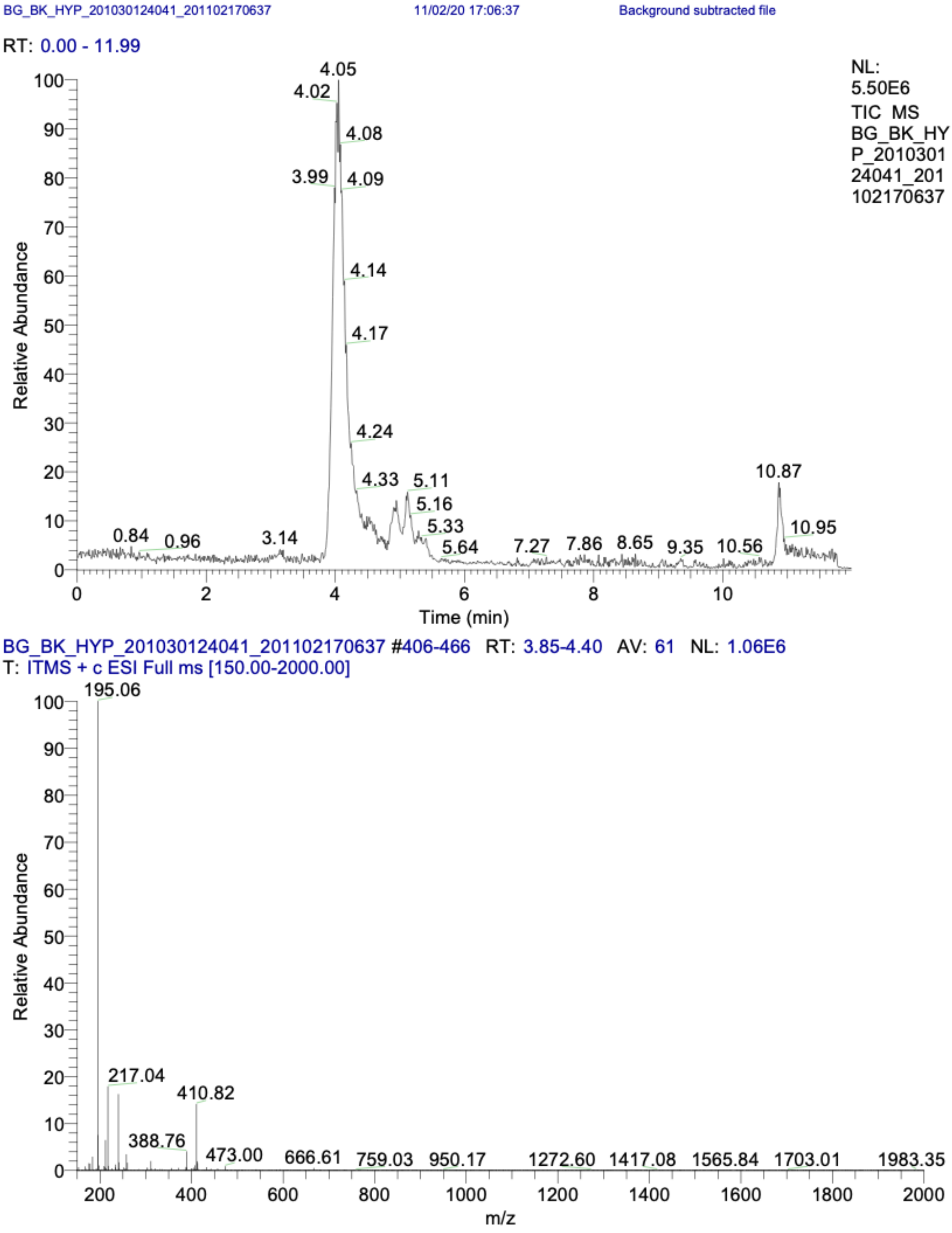

Supplementary Figure 52: LCMS-data for compound HYP.

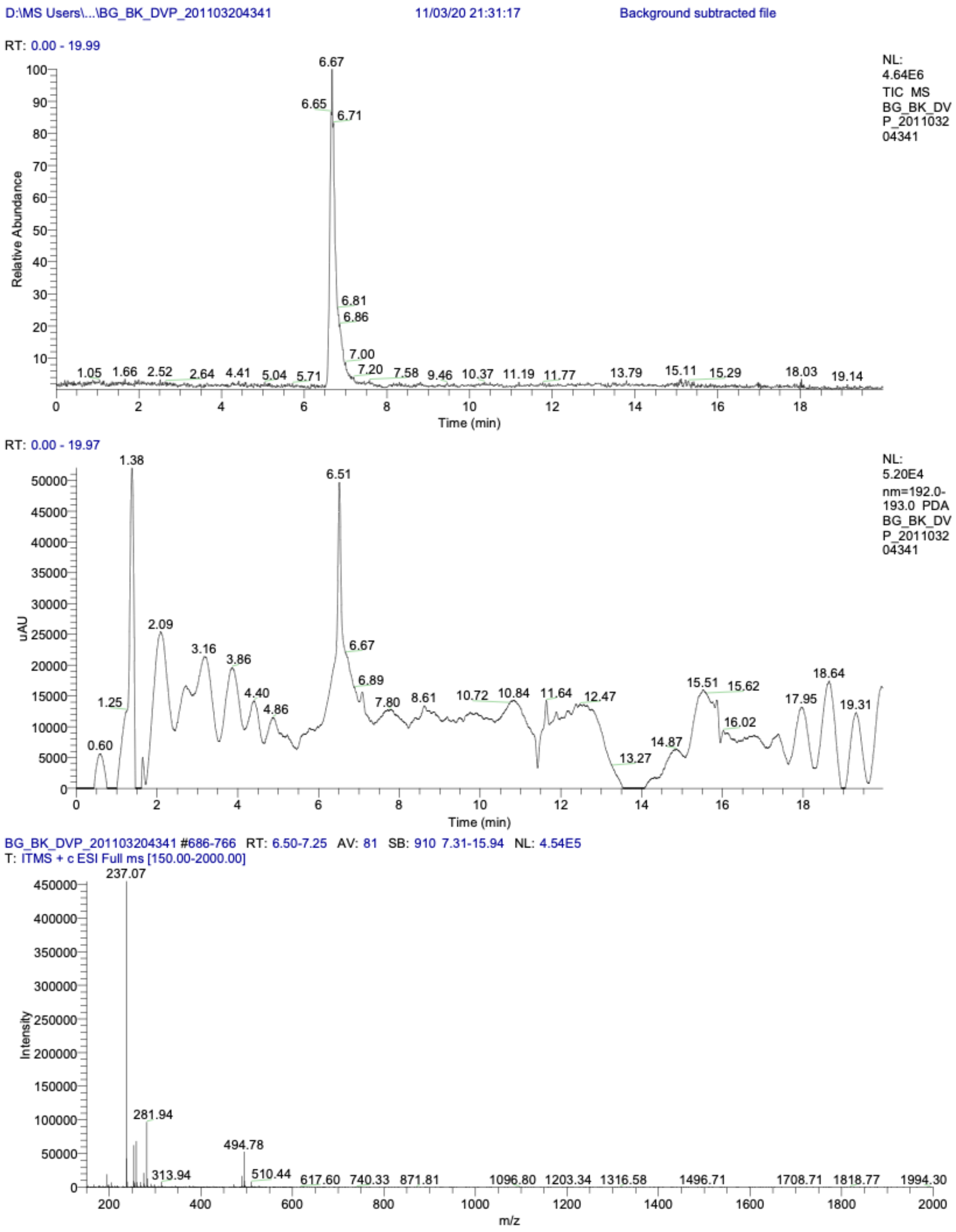

Supplementary Figure 53: LCMS-data for compound DVP.

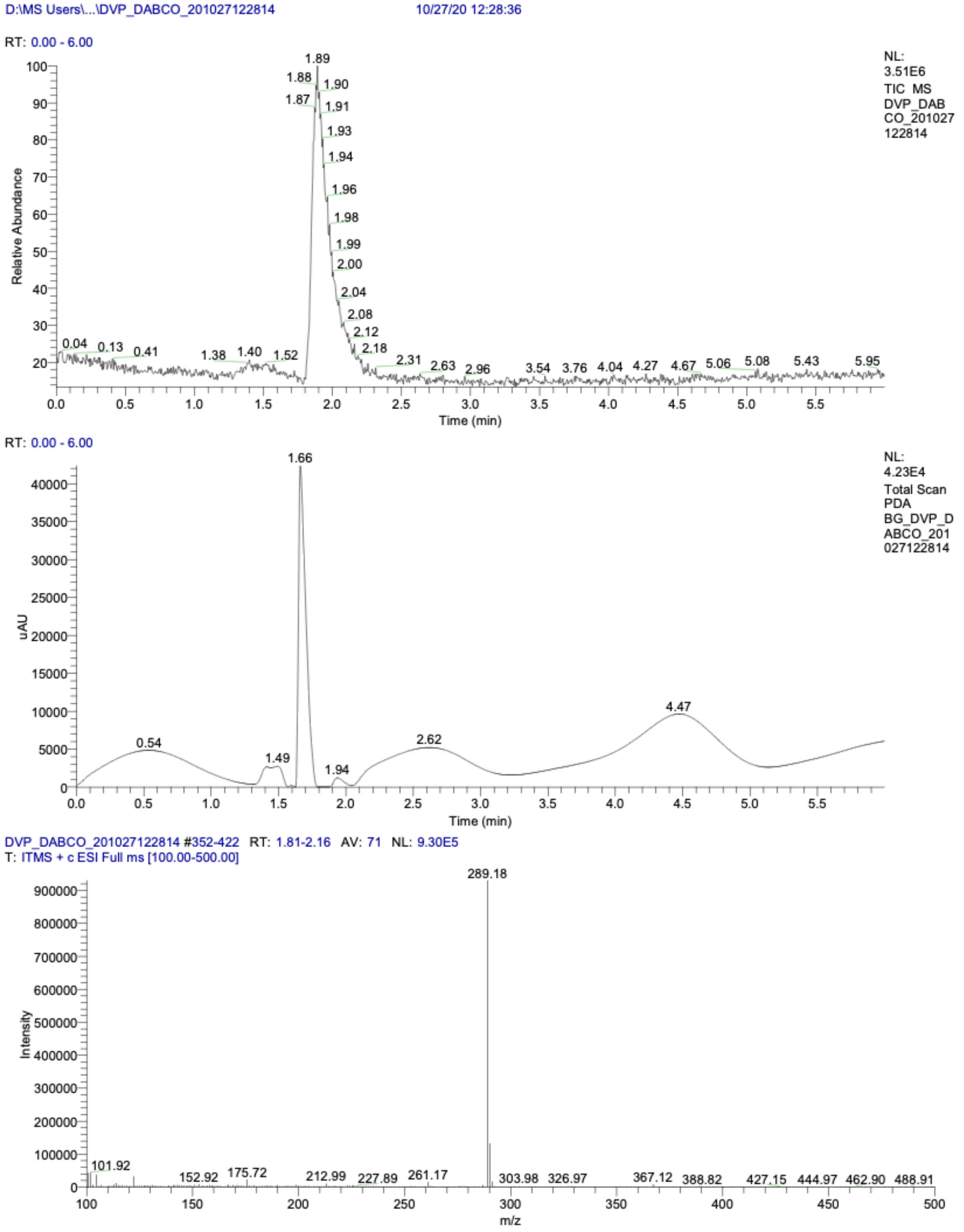

Supplementary Figure 54: LCMS-data for compound DVP-t-Am-1.

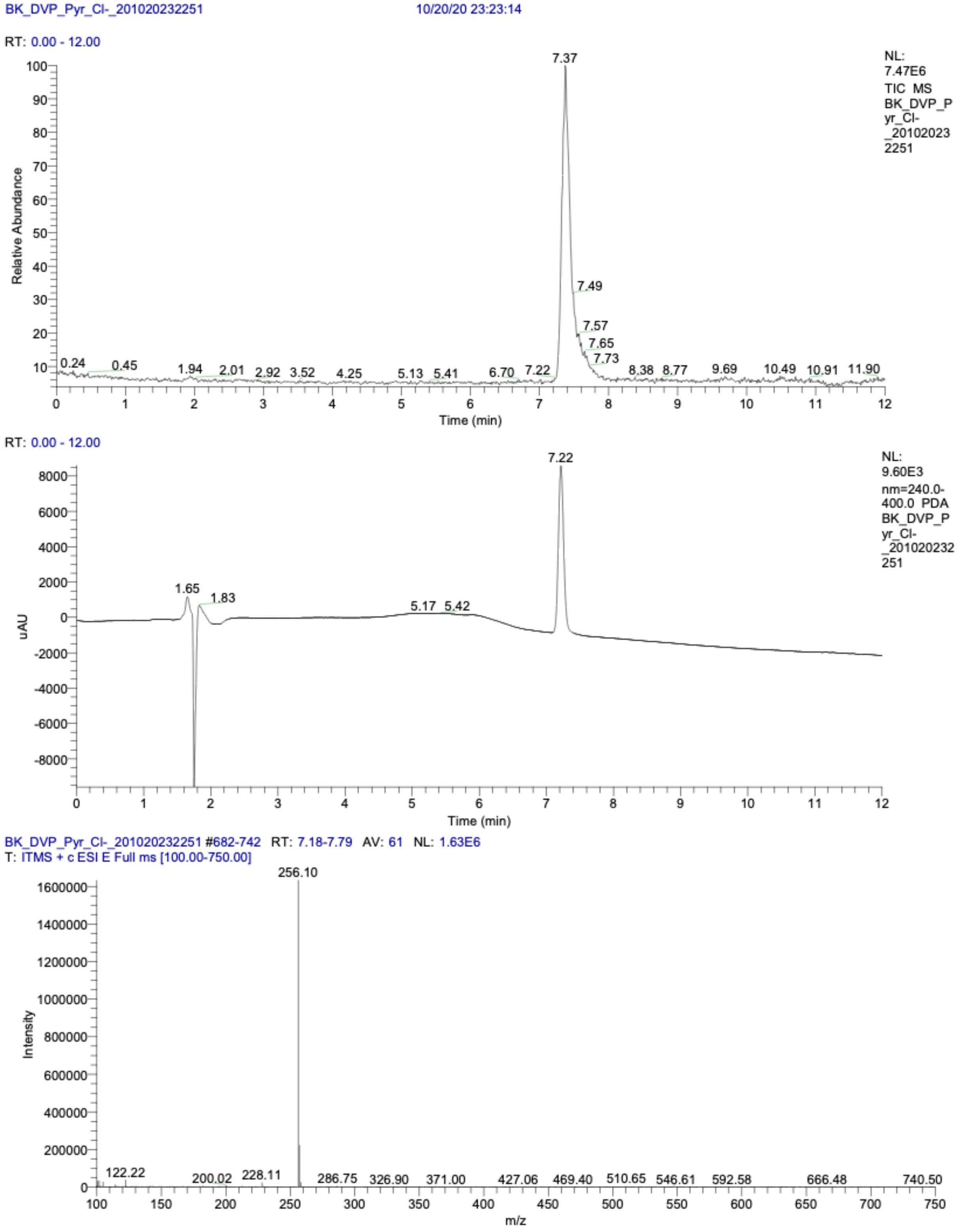

Supplementary Figure 55: LCMS-data for compound DVP-t-Am-2.

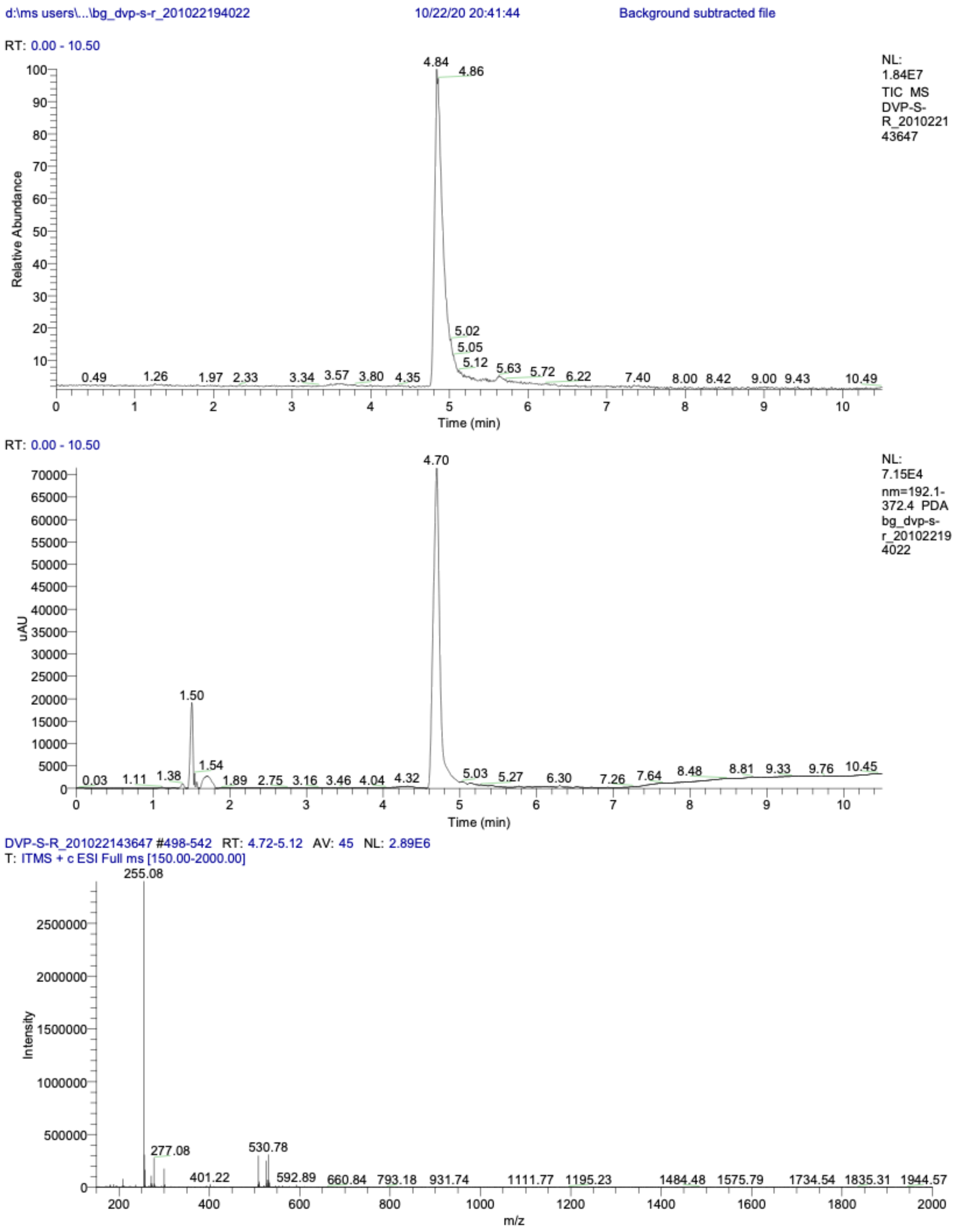

Supplementary Figure 56: LCMS-data for compound DVP-S.

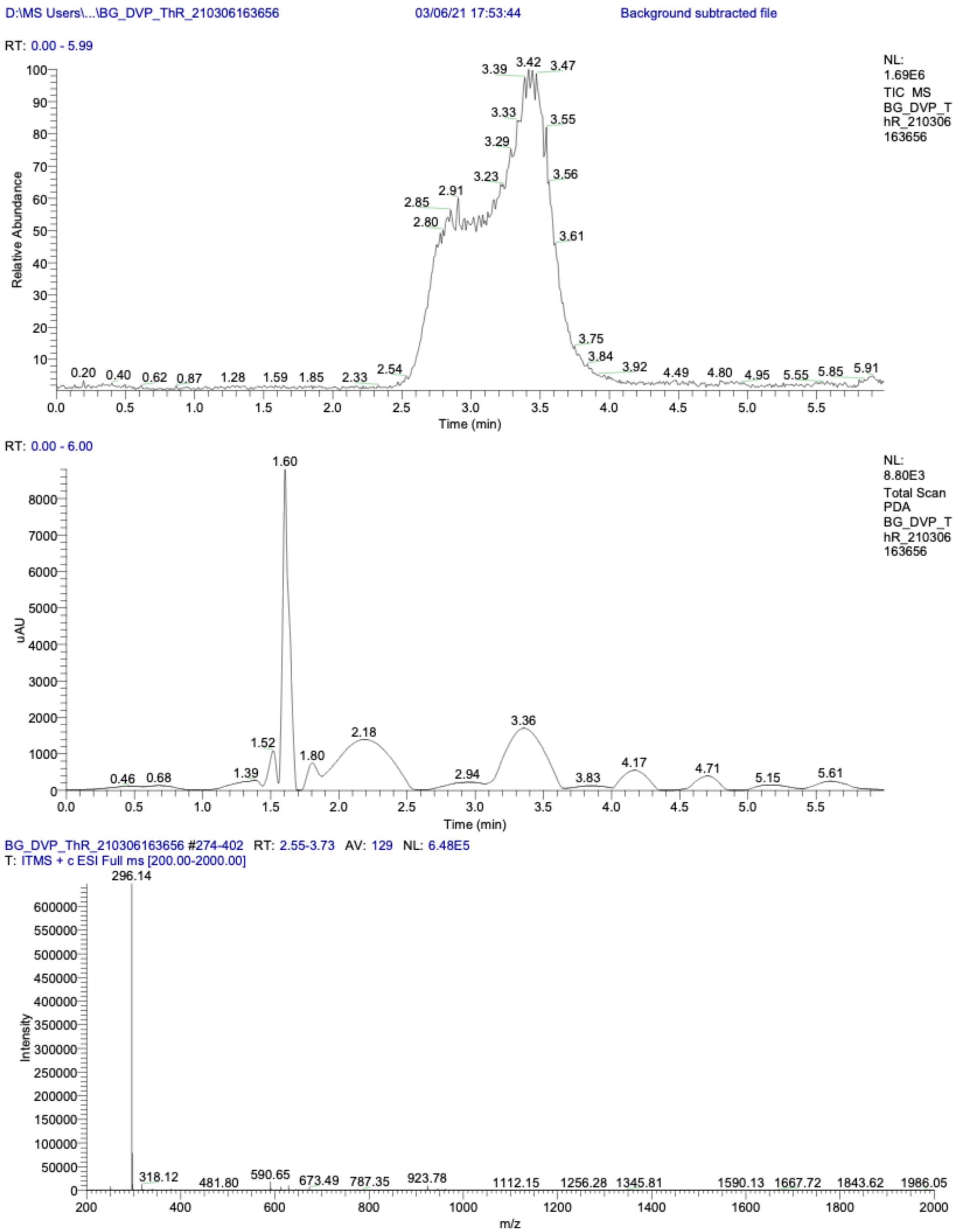

Supplementary Figure 57: LCMS-data for compound DVP-N.

Supplementary References

1. Rambaud, M., Vecchio, A. del & Villieras, J. Wittig-Horner Reaction in Heterogenous Media: V1. An Efficient Synthesis of Alkene-Phosphonates and αL-Hydroxymethyl-α-Vinyl Phosphonate in Water in the Presence of Potassium Carbonate. *Synth. Commun.* **14**, 833–841 (1984).
2. Garzon, C., Attolini, M. & Maffei, M. Synthesis of β-aminovinylphosphonates by organocatalytic nucleophilic displacement of acetate with amines. *Tetrahedron Lett.* **51**, 3772–3774 (2010).
